# The health outcomes of shelter and settlements interventions in humanitarian crises: a systematic review

**DOI:** 10.1186/s13031-026-00811-3

**Published:** 2026-06-20

**Authors:** Elizabeth Jarman, Lundi-Anne Omam, Jayshree Bagaria, Lizzie Babister, Jim Kennedy, Susannah Webb, Eba Pasha, Charles Kelly

**Affiliations:** 1 Independent Researcher, Nairobi, Kenya; 2 Queen’s University Belfast, Center for Public Health, Belfast, UK; 3 Cambridge Public Health Interdisciplinary Research Centre, Department of Psychiatry, University of Cambridge, Cambridge, Cambridgeshire, UK; 4 Independent Researcher, Edinburg, UK; 5 Habitat for Humanity International, Americus, US; 6 Global Shelter, Land and Site Coordination Cluster, Geneva, Switzerland; 7 CARE International UK, London, UK; 8 Global Health Cluster, World Health Organization, Geneva, Switzerland

**Keywords:** Shelter, Settlement, Humanitarian crisis, Health outcomes

## Abstract

**Background:**

Shelter and settlement are critical determinants of health in humanitarian crises, yet their impacts on health outcomes remain underexplored. This review synthesizes published evidence on how shelter and settlement interventions impact health outcomes in humanitarian crises.

**Methods:**

A systematic review of peer-reviewed literature was conducted to identify studies examining the association between shelter and settlement interventions and health outcomes in humanitarian crises. Following the Preferred Reporting Items for Systematic Reviews and Meta-Analyses guidelines a protocol was developed and three databases were searched for studies published between 2005 and 2025. Relevant data were extracted and thematically analysed.

**Results:**

A total of 138 articles were found. Thematic analysis identified six themes (living conditions, accommodation type, camp and site planning, displacement status, essential household items and cash-based interventions). Three cross cutting themes of gender, climate change and vulnerability were also identified. Two themes (living conditions and accommodation type) are presented here. Poor living conditions including damaged shelters, poor housing design and materials were associated with increased risks of communicable diseases such as malaria, diarrhoea, and soil-transmitted helminth infections while overcrowding heightened injury risk and psychological distress. Accommodation type influenced health outcomes with short-term shelters linked to acute mental health challenges and increased injuries; medium-term shelters with increased risk of non-communicable disease; and long-term housing with deteriorations in chronic disease management.

**Conclusions:**

This systematic review synthesises the evidence base showing how shelter and settlement interventions play a role in influencing health outcomes in humanitarian crises. It shows that shelter is a critical yet often under recognised determinant of health in these settings. Health outcomes are influenced by housing quality, accommodation type and duration, camp and settlement planning, displacement dynamics including frequency and length of displacement, and access to essential household items. Poor shelter conditions are likely to increase the risk of communicable diseases and mental health issues and have the potential to worsen chronic disease outcomes and risk. A key contribution of this review is the identification of shelter as a core entry point for public health action, highlighting the need for clearer operational guidance on shelter typologies, minimum standards, and their integration with health, protection and social services.

**Supplementary Information:**

The online version contains supplementary material available at https://doi.org/10.1186/s13031-026-00811-3.

## Background

Humanitarian crises, defined as events that overwhelm local capacity and require assistance to support affected populations. They are generally classified as either major emergencies or complex humanitarian emergencies. Major emergencies involve disasters without political instability or armed conflict, whereas complex humanitarian emergencies are driven by political factors and are characterized by instability and armed conflict [1, 2]. Current global trends show a rising frequency of such humanitarian crises, with record numbers of people displaced from their homes, as a consequence of protracted armed conflicts, and climate-related disasters [3]. In 2025, an estimated 305 million people worldwide are reported to require urgent humanitarian assistance and protection. The Southern and Eastern Africa region hosts the largest number of people in need (85 million people). The Middle East and North Africa follow closely, with an estimated 59 million people in need of assistance [4].

The direct health impacts of humanitarian crises are well documented and vary over time. Trauma and injuries are typical in the immediate aftermath of a conflict or following a climate-related disaster, with outbreaks of communicable diseases in the short to medium term [5, 6]. They affect both those with chronic conditions, such an non-communicable disease, and lead to acute illnesses [2]. Humanitarian crises affect almost every aspect of health. As health problems are exacerbated by the crises, the health systems become disrupted, damaged and overwhelmed [6]. During humanitarian crises, the provision of shelter and formation of temporary settlements is one of the first pillars of an emergency response, providing physical protection and also acting as an important contributor to health, both mental and physical health, well-being, dignity and privacy [7, 8].

Shelter interventions include emergency shelters, transitional shelters, temporary tents, prefabricated shelters, container housing, cash-for-rent schemes and host-family support. These are accompanied by site planning considerations, local construction advice and essential household item (EHI) distributions [8] The most common reported risk factors for post disaster infectious disease outbreaks related specifically to shelter and settlements, include overcrowding and poor shelter [9]. Frameworks for shelters and settlement interventions, in humanitarian crises do exist, such as the International Federation of the Red Cross and Red Crescent, post-disaster shelter model however, these have large levels of overlap between the different time frames [7, 10]. The variability of shelter types, and the recurrent use of short-term shelters or medium-term shelters being used for longer than what would be recommended, meant categorisation by days, months or years living in the shelter was challenging. Therefore, they were categorised by short-term, medium-term and long-term shelters, as defined in Table 1.

**Table 1:** Accommodation types included in short term, medium term and long term categorisation within this paper

Shelter types	Definition
Short term	These are tents, community shelters, containers, and emergency housing
Medium term	Temporary trailer homes, prefabricated units/Emergency Management Agency (FEMA) trailers, rented accommodation, public-funded rental housing, public housing, new houses, disaster-recovery condominiums tents, concentrated rural settlements (CRS), temporary structures built on public land and retirement homes.
Long term	Rented or owned homes, public and private housing, disaster and restoration housing complexes, prefabricated units, and institutional settings like hospitals used as shelters or retirement homes, across urban, rural, and camp environments.

Whilst the links between shelter and healthcare are well documented, the evidence base on the indirect or systemic impacts of shelter interventions on health outcomes in humanitarian crises remains less developed [11]. This paper aims to help understand this gap by reviewing the literature on the impact of shelter and settlement interventions in humanitarian crises on health outcomes.

## Methodology

Following the Preferred Reporting Items for Systematic Reviews and Meta-Analyses (PRISMA) guidelines, a protocol was developed for this review between the end of March to 17 May 2025 by a team of three consultants and was reviewed by the steering committee for the Global Shelter Cluster (Appendix 1). We sought to examine the relationship between shelter and settlement interventions and health outcomes in humanitarian crises, in order to synthesise existing evidence, identify key gaps, and inform policy and practice.

The research question identified was how does shelter and settlement assistance influence health outcomes in humanitarian crises, and under what conditions do these impacts occur? Four further sub-questions were also developed and can be seen in Appendix 2. Inclusion criteria included papers with all three variables (humanitarian crises, shelter or settlement intervention and health outcomes) English language, articles published between January 2005 and April 2025 and peer-reviewed literature of primary research studies (observational studies, case reports, case series, case studies, retrospective studies, cohort studies). 2005 was chosen as the start of the search because 96% of humanitarian papers identified in previous studies on shelter and health outcomes were found to be from after 2005. In this review, we applied this definition across settings irrespective of country income level, including both low- middle-, and high-income contexts where events such as floods and earthquakes exceeded local response capacity. As such, high income settings were included where the scale of the emergency aligned with the definition of humanitarian crises. We also recognise that resource availability varies significantly across contexts; therefore, findings from higher-resource settings were interpreted with attention to contextual differences, with a view toward identifying transferable lessons relevant to lower-resource and humanitarian settings. Excluded were systematic reviews, commentaries, editorials, opinion pieces, general narrative reviews, conference abstracts and research protocols.

Three electronic databases were searched (PubMed, Embase, Medline) using Medical Subject Headings (MeSH) terms where available and searches were conducted for grey literature. Duplicates arising from searches across multiple databases were removed using EndNote (version 20). These were imported into Rayyan [12] where more duplications were removed. A two-phase screening process was conducted by two reviewers (EJ and LAO) to determine studies for inclusion, with screening of titles and abstracts followed by full text screening, using Rayyan. Data extraction was conducted by two reviewers (EJ and LAO). The quality of all included studies was assessed using tools appropriate to each study design (Newcastle-Ottawa Scale (NOS), Cochrane Risk of Bias Tool, Critical Appraisal Skills Programme (CASP) checklist, Flinders University checklist, which evaluates Authority, Accuracy, Coverage, Objectivity, Date, and Significance (AACODS). Publications were scored and assigned a number from 1 to 100. Publications were classified as high (80–100%), medium (60–79%), or low (< 60%).

The extracted data was categorised by study characteristics (Appendix 2). Data collected on shelter and settlement intervention and health outcome variables were reviewed and categorised into six emerging themes. These were documented using excel and summarised on PowerPoint for discussion with the steering group. Within each theme, the data was further grouped into sub-groups and data synthesised into tables.

Detailed methodology can be found in Appendix 3.

## Results

**Fig. 1:**
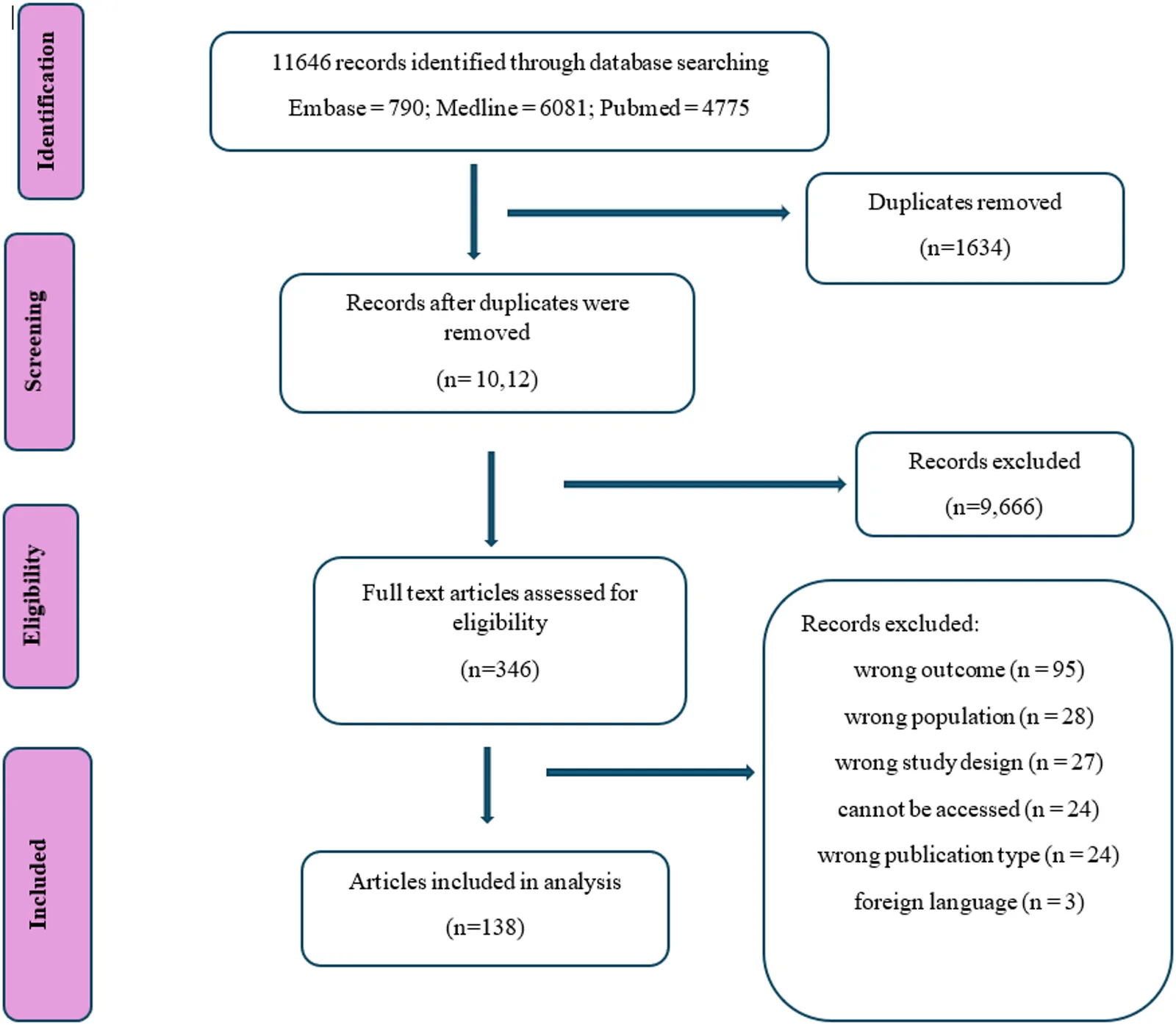
PRISMA chart showing selection process of included articles

The initial database search resulted in 11,646 articles. After eliminating duplicates, titles and abstracts, 10,012 articles were screened for relevance of study’s inclusion criteria. The full text of 346 articles was then assessed, with 138 articles selected for inclusion in the review (Fig. 1).

**Fig. 2:**
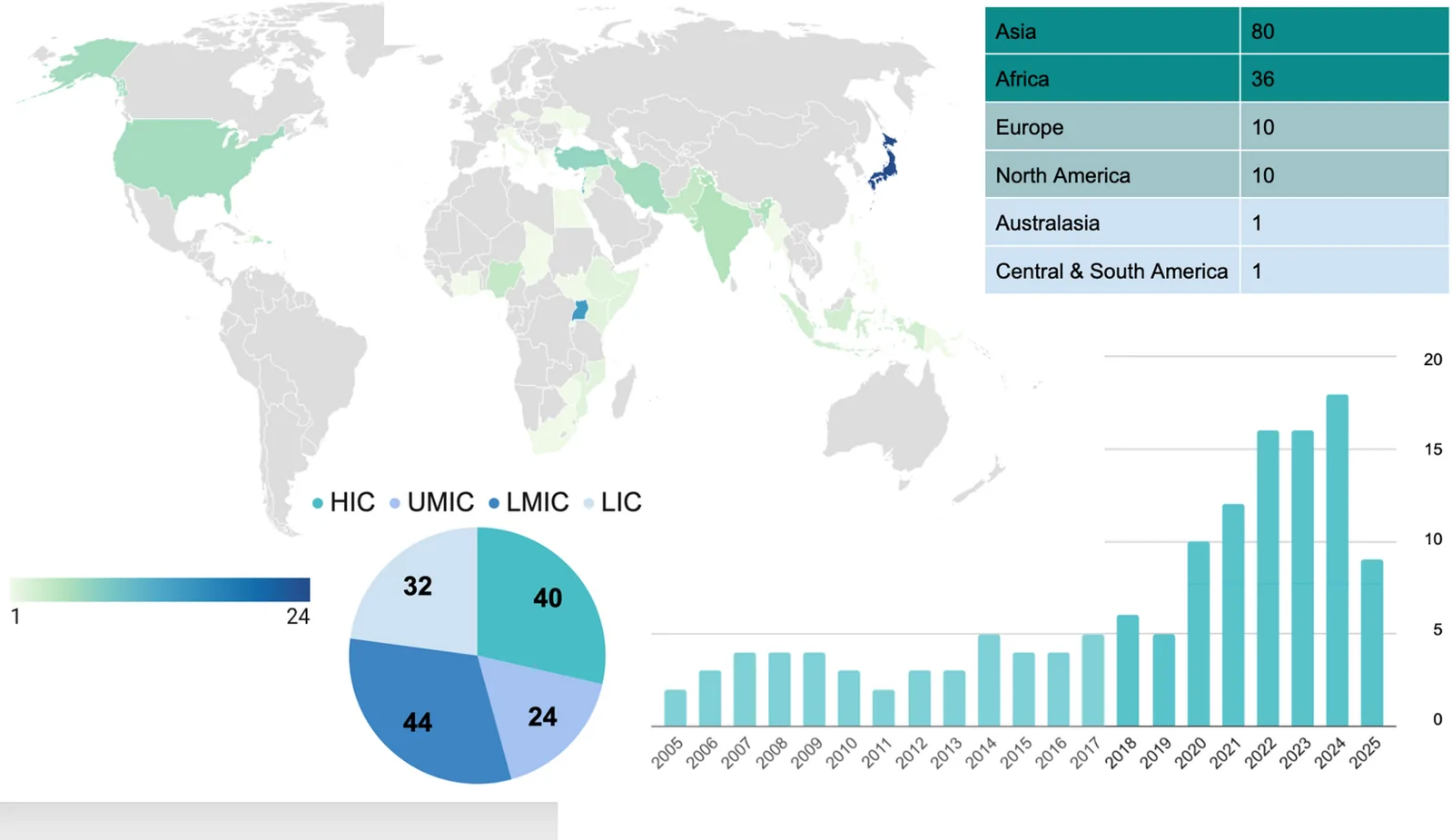
Geographical distribution, year of publication, region of interest and country income category of papers included in the studies

Most of the 138 papers included in the review (Appendix 2) were cross sectional studies (*n* = 79), followed by qualitative studies (*n* = 25) and cohort studies (*n* = 18). Nine studies utilised mixed methods study designs and one study was a randomised controlled trial (RCT). One third were studies from countries affected by earthquakes (*n* = 42). Fifty-three articles referred to a displacement crisis and 37 to armed conflict. The rest were predominantly meteorological disasters, with a handful on food insecurity and epidemics. Most papers were from Asia (*n* = 80), and Africa (*n* = 36) and most were published between 2020 and 2024. Forty (*n* = 40) studies were from high income countries (HICs), 24 from upper middle-income countries (UMIC), 44 from LMICs and 32 from low-income countries (LIC) (Fig. 2).

Seventy-four (*n* = 74) studies included primary data on mental health, followed by communicable diseases (*n* = 41), NCDs (*n* = 30), nutrition (*n* = 13), burns and injuries (*n* = 10), sexual and reproductive health (SRH) (*n* = 5) and gender-based violence (GBV) (*n* = 5). Health outcomes were measured mostly by a change in score (*n* = 52) or case numbers (*n* = 37).

Of 138 papers included in this review, the reviewers rated 109 papers as high quality, 12 as medium quality, 2 as good quality and 13 as low-quality papers. Two papers on economic evaluations were not rated. Six emergent themes were identified: (i) living conditions, (ii) accommodation type, (iii) camp and site planning, (iv) displacement status, (v) Essential Household Items (EHIs), and (vi) cash-based interventions and housing assistance. In addition, there were three cross cutting themes identified: (a) gender, (b) climate change and (c) vulnerability. Further details on the study characteristics of the 138 papers can be found in Table 2. For the scope of this paper^1^, the findings related to Themes I and II will be presented in the following sections.

**Table 2:** Study Characteristics

Study type		
Cross Sectional	Albelbeisi [13]; Albelbeisi [14], Albelbeisi [15]; Kibone [16]; Alduraidi [17]; ul Haq [18]; Ide-Okochi [19]; Ide-Okochi [20]; Namubiru [21]; Familiar [22]; Nakano [23]; Habib [24]; Thapa [25]; Borah and Saika [26]; Kun [27]; El-Ghitany [28]; Erdoğan [28]; Shabani [29]; Ugbe [30]; Seidi [31]; Mazhak [32]; Akaishi [33]; Kar [34]; Balakrishnan [35]; Segal [36]; Jinnouchi [37]; Horikoshi [38]; Suzuki [39]; Van Zandvoort [40]; van Berlaer [41]; Lett [42]; Yadav [43]; Biswas [44]; Kuwabara [45]; Balasuriya [46]; Kinoshita et al. [47]; Deka et al. [48]; Pham [49]; Tay et al. [50]; Di Marcantonio [51]; Akinyemi [52]; Math et al. [53]; Semakula et al. [54]; Bilgehan et al. [55]; Kaplan [56]; Wang [57]; Oboth [58]; Kuniyoshi et al. [59]; Klabbers [60]; Al-Khatib [61]; Patel [62]; Searle [63]; Searle [64]; Ejembi [65]; Andersen [66]; Abramson [67]; Laurito et al. [68]; Yaksi [69]; Roberts [70]; Ahmed [71]; Matsubara et al. [72]; Matsubara et al. [73]; Lu [74]; Suzuki [75]; Seruwagi [76]; Almasalkhi [77]; Abass [78]; Simeone et al. [79]; Ay Kesgin [80]; Salcoğlu [81]; Irmansyah [82]; Ylade et al. [83]; Kuniyoshi et al. [59]; Khan et al. [84]; Dræbel et al. [85]; Muir et al. [86]; Doocy [87]; Chan [88]; Sitwat [89]; Esfandyari [90]; Presser [91]	81
Qualitative methods	Hamamra [92]; Cardoso [93]; Khai [94]; MirMohamadali [95]; Chowdry [96]; Veronese [97]; Sabah [98]; Klabbers [99]; Benoni [100]; Kemei [101]; Logie [102]; Wu [103]; Abi Chahine [104]; Giusti [105]; Ruggiero [106]; Ekezie [107]; Andrade [108]; Brunson [109]; Richardson [110]; Andrade Pérez-Sales [111]; Chiumento [112]; Lee [113]; Mori [114]; Ishii [115]	24
Cohort studies	Takahashi [116]; Nagao [117]; Smeeth [118]; Morishima [119]; Basaran [120]; Sasaki [121]; Kawakami [122]; Yazawa [123]; Nöthling [124]; Goenjian [125]; Burns [126]; Tanji [127]; Kloos [128]; O’Laughlin [129]; Nagata [130]; Lin [131]	16
Mixed Methods	Zaman [132]; Karaki [133]; Abu Ibaid [134]; Jabali [135]; Nabulsi [136]; Falb [137]; Rasmussen [138]; Balakrishnan [35]; Nasar [139]; Goenjian [125]	10
Economic studies	Howard [140]; Orach [141]	2
Case reports	Yee [142]; Brandenburg [143]; Ayoya [144]	3
Case control	Takarinda [145]; Yzermans [146]	2
RCT	Kimani [147]	1

Types of Crises		
Armed conflict	Albelbeisi [13]; Albelbeisi [14], Albelbeisi [15], Hamamra [92]; Cardoso [93]; Thepa [25]; Smeeth [118]; Mazhak [32]; Veronese [97]; van Berlaer [41]; Nasar [139]; Sabah [98]; Benoni [100]; Tay [50]; Lee [113]; Al-Khatib [61]; Falb [137]; Almasalkhi [77]; Khai [94]; Ay Kesgin [80]; Seruwagi [76]; Ekezie [107]; Ahmed [71]; Roberts [70]; Abi Chahine [104]; Patel [62]; Logie [102]; Oboth [58]; Dræbel et al. [85]; Kemei [101]; Akinyemi [52]; Pham [49]; Jabali [135]; Familiar [22]; Ugbe [30]; Takarinda [145]; Burns [126]; Lett [42]	37
Displacement	Kibone [16]; Alduraidi [17]; Namubiru [21]; Familiar [22]; Zaman [132]; Habib [24]; El-Ghitant [28]; Ugbe [30]; Karaki [133]; Seidi [31]; Takarinda [145]; Abu Ibaid [134]; Segal [36]; Van Zandvoort [40]; Pérez-Sales [111]; Burns [126]; Lett [42]; Jabali [135]; Nasar [139]; Pham [49]; Marcantonio [51]; Akinyemi [52]; Howard [140]; Orach [141]; Semakula et al. [54]; Khan et al. [84]; Kemei [101]; Dræbel et al. [85]; Oboth [58]; Logie [102]; Klabbers [60]; Klabbers [99]; O’Laughlin [129]; Patel [62]; Ejembi (2018) [65]; Andersen [66]; Nabulsi [136]; Abi Chahine [104]; Roberts [70]; Ahmed [71]; Ekezie [107]; Seruwagi [76]; Rasmussen [138]; Ay Kesgin [80]; Chiumento [112]; Richardson [110]; Kimani [147]; Cardoso [93]; Tay [50]; Lee [113]; Al-Khatib [61]; Almasalkhi [77]; Presser [91]	53
Earthquake	Ide-Okochi [19]; Takahashi [116]; Nakano [23]; Kun [27] Nagao [117]; Erdoğan [28]; MirMohamadali [95]; Ide-Okochi [20]; Morishima [119]; Jinnouchi [37]; Horikoshi [38]; Suzuki [39]; Basaran [120]; Tanji [127]; Kuwabara [45]; Kinoshita [47]; Bilgehan et al. [55]; Sasaki [121]; Esfandyari [90]; Kaplan [56]; Wang [57]; Kuniyoshi et al. [59]; Mori [114]; Kawakami [122]; Nagata [130]; Yazawa [123]; Ishii [115]; Ayoyo [144]; Laurito [68]; Yaksi [69]; Giusti [105]; Matsubara et al. [72]; Matsubara et al. [73]; Chan [88]; Lu [74]; Suzuki [75]; Goenjian [125]; Brunson [109]; ¸Salcoğlu [81]; Doocy [87]; Irmansyah [82];	42
Flood	Yadav [43]; Biswas [44]; Balasuriya [46]; Deka et al. [48]; Lin [131]; Sitwat [89]; Wu [103]; Nöthling [124]; Abass [78];ul Haq [18]; Borah and Saika [26]; Shabani [29]	12
Extreme weather event	Akaishi [33]; Math et al. [53]; Yee [142]; Abramson [67]; Brandenberg [143]; Kloos [128]; Ruggiero [106]; Andrade [108]; Ylade et al. [83]; Simeone et al. [79]; Kar [34]; Chowdry [96]; Searle [64]; Balakrishnan [35]; Andrade [111]; Searle [63]; Klabbers [60]; Nakano [23]; Horikoshi [38]; Kawakami [122]; Yazawa [123]; Laurito [68]; Matsubara et al. [72]; Matsubara et al. [73]; Irmansyah [82]	25
Epidemic	Khai [94]; Klabbers [99]; Ylade et al. [83]; Nasar [139]	4
Volcano	Muir et al. [86]	1
Explosion	Yzermans [146]	1

Shelter and settlement		
Direct	Albelbeisi [13]; Albelbeisi [14], Albelbeisi [15]; Kibone [16]; Alduraidi, H(2024); ul Haq [18]; Ide-Okochi, [19]; Namubiru [21]; Familiar [22]; Zaman [132]; Takahashi [116]; Hamamra [92]; Nakano [23]; Cardoso [93]; Habib [24]; Thapa, S [25]; Borah and Saika [26]; Kun [27]; Nagao [117]; El-Ghitant [28]; Erdoğan [28]; Shabani [29]; Khai [94]; Ugbe [30]; Seidi [31]; Smeeth [118]; Karaki [133]; MirMohamadali [95]; Takarinda [145]; Mazhak [32]; Abu Ibaid [134]; Akaishi [33]; Ide-Okochi [20]; Kar [34]; Balakrishnan, A K [35]; Morishima [119]; Segal [36]; Chowdry [96]; Veronese [97]; Jinnouchi [37]; Horikoshi [38]; Yzermans [146]; Suzuki [39]; van Zandvoort [40]; Pérez-Sales [111]; Basaran [120]; Presser [91]; van Berlaer [41]; Burns [126]; Lett [42]; Jabali [135]; Yadav [43]; Tanji [127]; Nasar [139]; Biswas [44]; Kuwabara [45]; Sabah [98]; Balasuriya [46]; Klabbers [99]; Kinoshita [47]; Andrade [108]; Benoni [100]; Deka et al. [48]; Pham [49]; Tay [50]; Marcantonio [51]; Akinyemi [52]; Math et al. [53]; Howard [140]; Orach [141]; Semakula et al. [54]; Bilgehan et al. [55]; Sasaki [121]; Khan et al. [84]; Yee [142]; Esfandyari [90]; Lee [113]; Kaplan [56]; Kemei [101]; Lin [131]; Wang [57]; Dræbel et al. [85]; Oboth, [58]; Kuniyoshi et al. [59]; Logie [102]; Klabbers [60]; Mori [114]; Al-Khatib [61]; Kawakami [122]; Muir et al. [86]; Nagata [130]; O’Laughlin [129]; Patel [62]; Yazawa [123]; Searle [63]; Searle [64]; Ejembi, J (2018) [65]; Andersen [66]; Ishii [115]; Abramson [67]; Brandenberg [143]; Sitwat [89]; Ayoyo [144]; Wu, J [103]; Kloos [128]; Laurito [68]; Yaksi [69]; Nabulsi [136]; Abi Chahine [104]; Roberts [70]; Giusti [105]; Ahmed [71]; Matsubara et al. [72]; Matsubara et al. [73]; Ruggiero [106]; Ekezie [107]; Nöthling [124]; Ylade et al. [83]; Abass [78]; Simeone et al. [79]; Ay Kesgin [80]; Chiumento [112]; Brunson [109]; Richardson [110]; Salcoğlu [81]; Kimani [147]; Doocy [87]; Irmansyah [82]	125
Process	Sitwat [89]; Ayoyo [144]; Wu, J [103]; Kloos [128]; Laurito [68]; Chan [88]; Ekezie [107]	7
Indirect	Karaki [133]; Ide-Okochi [19]; Laurito [68]; Yaksi [69]; Nabulsi [136]; Abi Chahine [104]; Roberts [70]; Giusti [105]; Ahmed [71]; Matsubara et al. [72]; Matsubara et al. [73]; Ruggiero [106]; Andrade [111]; Falb [137]; Lu [74]; Suzuki [39]; Seruwagi [76]; Almasalkhi [77]; Rasmussen [138]; Nöthling [124]; Ylade et al. [83]; Abass [78]; Simeone et al. [79]; Ay Kesgin [80]; Goenjian [125]	25

Health Sector		
Communicable	Kibone [16]; ul Haq [18]; Takarinda [145]; Burns [126]; Klabbers [99]; Deka et al. [48]; Howard [140]; Semakula et al. [54]; Khan et al. [84];; Yee [142]; Dræbel et al. [85]; Logie [102]; Klabbers [60]; O’Laughlin [129]; Patel [62]; Searle [63]; Searle [64]; Ejembi, J (2018) [65]; Ishii [115]; Ahmed [71]; Richardson [110]; Kimani [147]; Lee [113]; van Berlaer [41]; Khai [94]; Balasuriya [46]; Lin [131]; Karaki [133]; Balakrishnan [35]; Yadav [43]; Kuniyoshi et al. [59]; Abi Chahine [104]; Wu, J [103]; Oboth [58]; van Zandvoort [40]; Kemei [101]; Roberts [70]; Chan [88]; Habib [24]; Al-Khatib [61]; Sabah [98];	41
Mental Health	Lee [113]; van Berlaer [41]; Karaki [133]; Balakrishnan [35]; Yadav [43]; Kuniyoshi et al. [59]; Abi Chahine [104]; Albelbeisi [13]; Alduraidi [17]; Hamamra [92]; Thapa [25]; Borah and Saika [26]; Kun [27]; El-Ghitant [28]; Shabani [29]; Ugbe [30]; Seidi [31]; Smeeth [118]; Mazhak [32]; Akaishi [33]; Ide-Okochi [20]; Kar [34]; Morishima [119]; Segal [36]; Veronese [97]; Horikoshi [38]; Yzermans [146]; Suzuki [39]; Pérez-Sales [111]; Tanji [127]; Nasar [139]; Kuwabara [45]; Kinoshita [47]; Pham [49]; Akinyemi [52]; Math et al. [53]; Sasaki [121]; Wang [57]; Kawakami [122]; Muir et al. [86]; Yazawa [123]; Abramson [67]; Sitwat [89]; Kloos [128]; Laurito [68]; Yaksi [69]; Matsubara et al. [72]; Matsubara et al. [73]; Ruggiero [106]; Lu [74]; Suzuki [75]; Seruwagi [76]; Rasmussen [138]; Nöthling [124]; Ylade et al. [83]; Abass [78]; Simeone et al. [79]; Goenjian [125]; Şalcoğlu [81], Irmansyah [82]; Familiar [22]; Nagata [130]; Almasalkhi [77]; Roberts [70]; Falb [137]; Chan [88]; Jabali [135]; Benoni [100]; Tay [50]; Andrade [111]; Habib [24]; Al-Khatib [61]; Kaplan [56].	74
NCDs	van Berlaer [41]; Khai [94]; Balasuriya [46]; Lin [131]; Karaki [133]; Balakrishnan [35]; Yadav [43]; Kuniyoshi et al. [59]; Abi Chahine [104]; Wu, J [103]; Andrade [108]; Nagata [130]; Almasalkhi [77]; Roberts [70]; Ide-Okochi [19]; Zaman [132]; Takahashi [116]; Nakano [23]; Nagao [117]; Jinnouchi [37]; Bilgehan et al. [55]; Mori [114]; Andersen [66]; Chan [88]; Jabali [135]; Tay [50]; Andrade [111]; Habib [24]; Al-Khatib [61]; Sabah [98]	30
Injuries and burns	van Berlaer [41]; Biswas [44]; Abu Ibaid [134]; Basaran [120]; Presser; Lett [42]; Brandenberg [143], Doocy [87]; Kemei [101]; Andrade [108]	10
Nutrition	van Berlaer [41]; Oboth [58]; Falb [137]; Albelbeisi [13]; Albelbeisi [15]; Namubiru [21]; Erdoğan [28]; MirMohamadali [95]; Marcantonio [51]; Ayoyo [144]; Nabulsi [136]; Ay Kesgin [80]; Esfandyari [90];	13
SRH	Wu, J [103]; Esfandyari [90]; Orach [141]; Giusti [105]; Brunson [109]; Chowdry [96]	5
GBV	Chowdry [96]; Familiar [22]; Biswas [44]; Chiumento [112]; Cardoso [93].	5

### Theme 1 - living conditions

Data analysis identified ‘living conditions’ as an important overarching theme. Forty publications related to the impact of living conditions on health outcomes following a humanitarian crises were identified and data was sub-divided into three themes; housing conditions; overcrowding and energy.

#### Housing conditions

Twenty papers reviewed showed that housing conditions impacted upon on communicable disease risk (*n* = 6), mental health (*n* = 8), injuries (*n* = 1), GBV (*n* = 1) NCDs (*n* = 1). For communicable diseases, three articles considered the impact of building materials used, whilst the other three reviewed impact of property damage. Children living in mud houses were found to be more at risk of diarrhoea, those in flood prone houses had higher risk of soil-transmitted helminths (STHs) and children living in thatch houses were at markedly greater risk of contracting malaria. Houses that leaked water, or had holes in their eaves also increased the risk of contracting malaria [64, 145].

For malaria, one paper reported the odds of infection were over three times higher for those residing in a house with holes, open eaves or makeshift windows that could not be closed [145]. In one study, children living in households (HHs) with walls constructed of thatch and cardboard were four and a half times more likely to contract malaria compared to those living in houses built with brick and cement or mud. Children living in HHs with thatch roofs were twice as likely to contract malaria compared to those with roofs constructed of tarpaulins and iron sheets [54]. Two studies from Mozambique noted that malaria prevalence was higher in HHs with significant damage or complete destruction from cyclones compared to those with no damage, and that living in a house with even minimal damage, resulted in lower use of insecticide treated nets (ITNs). This is likely due to a combination of factors including inadequate ownership of ITNs, affected in part by household damage, but also due to likely delays and interruptions to community ITN distribution plans [63, 64].

In camps in Lebanon, water leakage within the home was significantly associated with multi-morbidity, with those living in houses with water leakage nearly twice as likely to have three or more health reports^2^. This pattern was more pronounced among respondents living in houses with severe food insecurity [24]. In Gaza, oPt, many families reported that damaged housing infrastructure and limited access to repair was a primary factor contributing to poor health outcomes [98].

#### Overcrowding

This sub-theme looked at the link between overcrowding and health outcomes and included twelve papers focused on communicable diseases (*n* = 4), mental health (*n* = 3), general physical health (*n* = 2), injuries (*n* = 2), NCDs (*n* = 1) and nutrition (*n* = 1).

Articles on communicable diseases looked at diarrhoea, HIV, respiratory conditions and skin conditions. In Pakistan, HH size was one of a number of factors found to affect the risk of diarrhoea in flood affected areas. Children in large HHs (> 8 people) were 7 times more likely to have diarrhoea than children in small HHs (1–4 people) [9]. Overcrowding in camps in Europe was seen as a major barrier to effective management of diseases such as scabies [110]. In Uganda, lack of privacy from overcrowding was reported to prevent HIV self-testing [148]. For respiratory disease, a study from Somaliland didn’t show evidence of a link between HH size and self-reported pneumonia [40]. However, in contrast, a qualitative study in older Syrian refugee populations in Lebanon reported experiencing increased pulmonary diseases believed due to the spread of viruses in overcrowded and unheated homes [104].

The two papers reporting on general health, showed that those living in crowded HHs were more likely to report two or more poor health outcomes than homes without crowding [24, 135]. In the West Bank, oPt, participants who lived in HHs with more than six individuals reported the lowest mean physical health score, whilst HHs with four-six individuals reported the highest, showing better perceived physical health [135]. However, this is likely to be context specific, with other factors, such as household income levels or home-ownership status potentially having a higher impact.

Similarly, with regards to mental health, HHs with 4–6 people in the West Bank, oPt reported the best mental health scores, compared to those living in smaller or larger households [135]. IDPs living in overcrowded shelters in Nigeria had a higher risk of developing somatoform disorder [30] while refugees in Czechia who lived with strangers or lacked space experienced higher perceived stress [32].

#### Energy

The energy sub-theme includes provision of energy following an emergency. This includes electricity or liquified petroleum gas (LPG) gas, for use for cooking and heating depending on the context. Twelve papers looked at health outcomes related to energy, including the risk of injuries and burns and mental health implications.

Most papers found a negative association between loss of electricity or gas and mental health outcomes, including depression, emotional distress, anxiety, PTSD and major depressive episodes (MDE [72, 106, 111, 124]. Only one paper failed to find an association with PTSD, similarly with anxiety [73, 149]. Four papers examined the impact of heating supply on health outcomes in humanitarian crises. Access to heating in Türkiye within 72 h of the 2023 earthquake was associated with a lower risk of probable severe PTSD [69]. One study from Puerto Rico reported that lengthy power outages had a severe impact on mental health, as well as other significant long-term challenges, particularly regarding the refrigeration of essential medicines such as insulin [111]. In relation to heating methods, the use of electricity was identified as the safest option, while open and closed flame heating sources were associated with a higher incidence of injury [91].

### Theme 2 - accommodation type

A total of 30 publications reported on the impact of accommodation types on health outcomes among populations affected by humanitarian crises. Findings were classified by duration of displacement.

#### Short-term shelter

Twenty papers (appendix 3) focused on short-term shelter and settlements, with a duration of stay of weeks to months, and the health outcomes associated with them: mental health (*n* = 5), communicable diseases (*n* = 4), injuries and burns (*n* = 3),physical health (*n* = 3), NCDs (*n* = 3), nutrition (*n* = 2), access to services (*n* = 1), GBV (*n* = 1). In terms of mental health, three of five studies compared mental health outcomes across different types of short-term shelter while the other two looked at changes in mental health outcomes for those living in temporary accommodation. Three studies showed that temporary displacement was associated with a deterioration in mental health outcomes [13, 69, 150]. A study in Gaza, oPt found that IDPs residing in community shelters, such as schools, experienced better mental health outcomes compared to those living in tents [13]. Another study described an increased risk of PTSD in those living in temporary accommodation in China [27]. This finding is supported by one study that observed significantly higher rates of PTSD among individuals sheltering in tents compared to those in other forms of accommodation after the 2024 earthquake in Türkiye [150]. In contrast another study found no significant association between type of shelter and PTSD suggesting that other contextual or individual factors may influence the mental health impacts of displacement [69].

*In the USA*, an increase in communicable diseases was shown in those residing in community shelters, when compared to those displaced within the community after Hurricane Katrina [38]. Similarly, an outbreak of norovirus in shelter inhabitants amongst Ukrainian refugees was documented in Poland [113, 142]. Two further papers outlined management strategies of emergency shelters in relation to outbreak management, reflecting good practice and well-coordinated shelter response to control an outbreak [115, 151].

For injuries, two studies were conducted in refugee camps while one looked at management of an evacuation shelter in the USA.*al One paper* reported that, in Gulu, Uganda, in a population that was primarily (73%) displaced into temporary housing, 31% of deaths and 36% of disability injuries occurred in people’s dwellings. In Türkiye, an increase in burns was seen following an earthquake, with thje majority of burns taking place in the first week occurring in their own home. Thereafter the majority of burns took place in tents, with a larger proportion occurring outdoors. Electrical burns were most seen 46 days after the earthquake [120].

One study in Gaza, oPt, compared nutritional outcomes among individuals residing in tents, community shelters, and permanent housing. Interestingly, children living in permanent houses exhibited the highest rates of severe acute malnutrition (SAM) and moderate acute malnutrition (MAM), compared to those residing in schools (community shelter) or tents [15]. A study from Iran reported that women living in tents expressed concerns over the lack of safe and private locations for breastfeeding negatively impacting upon infant feeding practices [95].

Three papers reported on the management of short term, emergency shelters providing case reports or descriptions of good practice for shelter managers. *alone paper* described the medical response in emergency shelters following the Great East Japan Earthquake (GEJE) in 2011. In this response, contamination-control certified nurses visited shelters where unsanitary conditions were identified, offering guidance to improve hygiene and sanitation practices [115]. *Another study* [103] examined emergency sheltering practices in China, where pre-built and pre-allocated community shelters, most commonly located in schools, were equipped with water, food, gas, and electricity. These shelters were viewed as more stable alternatives to tents and enabled more efficient and concentrated health management, based upon lessons learned from the severe flooding in Anhui Province in 2016. Half of the shelter points had temporary medical points offering antenatal care, postpartum follow-up, and health guidance and management for the elderly and those living with chronic diseases. Pregnant women and newborns were prioritised for transfer to sites with better medical services such as mass shelters or county hospitals. Decisions on the location of shelter sites were made by township governments, in consultation with public health personnel [103, 115].

One paper examined access to healthcare for those living in tent cities in Türkiye which showed a significant decline in the proportion of participants able to meet their medication needs (50% decrease), access hospitals (60%), maintain NCD-specific nutrition (62%), and receive adequate social support (19%) after the earthquake compared to the pre-earthquake period [55].

For NCDs, a third of American interviewees, interviewed six weeks after the 2022 Eastern Kentucky floods, living in travel trailers, campers, cottages and campgrounds, reported their household experienced a worsening of allergies, high blood pressure, overall health, and lung conditions [152].

#### Medium-term shelter

Fourteen publications were related to medium-term shelters, predominantly in the aftermath of the GEJE of 2011: mental health (*n* = 10), NCDs (*n* = 3), general health (*n* = 1).

Among the seven studies comparing across shelter types, five examined prefabricated housing or containers, and two looked at disaster-recovery public condominiums, public housing, and rental housing. *Morishima et al.* noted an association between psychological distress and living in temporary housing. In addition, no association between privately rented temporary housing increases in sleep disturbance were found [60]. *Oone study* showed that residents of public housing (state-owned) and private housing in Japan for disaster survivors experienced higher rates of psychological distress and PTSD, with public housing also reporting increased insomnia [59]. *Another study* found that individuals living in temporary housing complexes had more visits to psychiatrists and physicians, and were more likely to seek help for MDE compared to the general disaster affected population [122].

Moreso, displacement to prefabs or disaster-recovery public condominiums in Japan was found to be associated with poorer mental health outcomes, including psychological distress, depressive symptoms, and anxiety when compared with those who remained in their own home, or were moved to other construction locations^3^[75, 119, 127]. Contrary to these findings, one study found no worsening in health scores five years after the disaster among those living in temporary prefabricated housing built on school grounds or in parks [153]. In China, relocation to concentrated rural settlements after an earthquake was found to be associated with a lower prevalence of PTSD symptoms compared to in-situ reconstruction [74]. In Türkiye, compared to tents, those living in prefabs had lower levels of somatisation, psychosis and paranoia, but higher levels of anxiety [154].

Three studies focused specifically on NCDs and related risk factors. *alone study* investigated the association between housing type and metabolic syndrome (MetS), finding that residents displaced to prefabricated units had higher rates of current smoking, low physical activity, and poor dietary intake than those who remained in their original homes [116]. Among individuals aged 65 years and older, there were significant differences in the incidence of MetS based on accommodation type, particularly regarding cholesterol levels, and obesity, though not for HbA1C or blood pressure. The increase in MetS was most notable for women [116]. Similarly, two papers found that individuals living in trailer-style temporary housing nine years after an earthquake had higher consumption of diets rich in alcoholic beverages, meat, and seafood compared to those who did not relocate. Initiation of smoking was also more common in this group, indicating that shelter type may influence long-term health behaviours [23, 123]. Two papers note increased rates of obesity and eczema in those living in prefabricated houses [59, 116].

#### Long-term shelter

Six papers looked at health outcomes in long-term shelters: mental health (*n* = 4), general health and NCDs (*n* = 3).

In terms of NCDs, in Japan, four years after the disaster, one study found that non-compliance with antihypertensive medication was twice as likely among those living in rental housing, public housing, and among individuals with poor self-rated health compared with those living in their own houses and was four times more likely among those residing in restoration public housing [19]. In Gaza, oPt, children living in privately owned houses had lower rates of anaemia compared to those living in rental houses [14].

Four studies focused on the impacts of different types of long-term shelter on psychological distress and mental health. In Japan, individuals living in rented housing, public housing, and public housing for disaster survivors were more likely to experience psychological distress [20]. In oPt, those living in their own houses and private flats experienced the best mean scores for mental health and psychological well-being [135].

One paper reviewed was a comparative analysis of mental health outcomes among three groups: individuals who were still displaced, those who had returned to their original homes, and those “in transition” was made. While returning home was associated with improved mental health relative to ongoing displacement, the best outcomes were observed among those who had relocated to new, stable accommodations [86]. Similarly, one study found that displaced individuals who remained in their school district had higher rates of PTSD compared with those who moved outside of their school district. There was no association seen with other mental health conditions [20].

## Discussion

The findings of this review highlight that the impacts of shelter interventions go well beyond its role in providing physical protection but also show that shelter is fundamental determinant of physical, mental, and social well-being. Across a wide range of humanitarian crises including displacement crises, armed conflict, earthquakes, extreme weather events and food insecurity, shelter and settlements consistently emerged as a critical factor influencing health outcomes. While this review included studies from high-income settings, we acknowledge that the transferability of findings to low-income and conflict-affected settings is not always straightforward. High-income countries often have stronger infrastructure, more robust health systems, and greater surge capacity, which shape both the design and effectiveness of shelter interventions. However, several cross-cutting lessons remain relevant across contexts. These include the importance of rapid shelter provision to reduce exposure-related morbidity, the integration of shelter with water, sanitation, and hygiene (WASH), nutrition and health services, attention to thermal comfort and ventilation, repurposing permanent structures like schools, hotels to provide improved shelter conditions, using prefabricated houses, and the need to prioritize vulnerable populations in shelter allocation. Additionally, approaches to coordination, standards setting, and the use of data for planning and monitoring offer useful insights, even if their implementation must be adapted to resource-constrained environments. We delve more into these lessons in the sections below.

### Housing conditions linked to communicable and mental health conditions

Inadequate living conditions, particularly overcrowding, poor housing conditions and poor infrastructure which expose individuals to extreme temperatures and unhygienic environments, contribute to heightened levels of stress anxiety, depression, and transmission of communicable diseases, injuries and burns.

In this review, communicable diseases were strongly associated with the physical condition and materials of shelters. Children living in mud or thatch homes compared to brick and iron, or in structures with damaged roofs or no windows faced higher risks of malaria and diarrhoea [9]. In flood-affected areas of India, poor-quality housing was also associated with increased risk of soil-transmitted helminth infections [18, 48], although *Deka et al.* found no significant link between flooring material and STH infection [48]. Our findings align with evidence from non-humanitarian crises, including the large-scale Mexican initiative to replace dirt floors with cement which has been shown to have meaningful health gains [155], *Rolfe et al.’s* findings that housing quality was significantly correlated with health outcomes [156], and *Adebowale et al.* who linked housing material with under five mortality, with a 46% higher risk of mortality for those who lived in houses made of inadequate housing materials [157]. These findings highlight the need for clearer operational guidance on shelter standards and typologies, particularly regarding materials, ventilation, and structural quality as core public health considerations. While evidence on specific components is mixed, improving basic shelter conditions appears to offer a relatively low-cost opportunity to reduce infectious disease risks, with potential health returns that warrant further cost-effectiveness analysis.

Overcrowding was found to be associated with a range of mental health conditions [30, 135]. Whilst optimal household size appears to be highly context specific, findings suggest that moderate household sizes may confer social and emotional support that benefit health outcomes, with very small and very large households presenting challenges. Overcrowding has important operational implications, highlighting the need for context-specific shelter standards that define acceptable occupancy levels and household typologies, while balancing space constraints with the potential social benefits of moderate household sizes to support mental well-being [158].

### Context specific programming

The relevance of the individual context to the impact on health of the shelter or settlement intervention was reported in this review. In resource limited settings such as Gaza, Turkey, Indonesia, people displaced to short term shelters and tents consistently displayed worse mental health outcomes compared with those displaced to more stable forms of emergency housing [13, 86, 150]. Evidence from Japan and USA showed that individuals in temporary or prefabricated housing experienced poorer health behaviours and increased long-term NCD risks, including metabolic syndrome, mental health and obesity [59, 116]. These findings suggest that the mental health impact of relocation depends on the context. In some settings, moving survivors away from the site of a disaster may reduce daily reminders of trauma and support psychological recovery, particularly in areas prone to repeated extreme events. However, evidence from the USA indicates that relocation to prefabricated housing can also be associated with poorer mental health outcomes, highlighting that the benefits of relocation are not guaranteed. The impact appears to depend on factors such as preservation of social networks, access to services, and the quality and design of housing, emphasizing the need for people-centred, context-adapted shelter strategies.

Furthermore, children residing in houses exhibited the highest rates for SAM, and MAM compared to those in schools or tents [15]. Children in houses may belong to households that are living in isolated or unsafe areas where food access, income generation and humanitarian reach are limited. Meanwhile, families in tents or schools may be closer to aid distribution points, referral pathways and community kitchens. These findings caution against treating tents as inherently better or worse. Instead, the context including type of emergency, location, access to services, protection risks, and livelihood opportunities, appear to be critical determinants of health outcomes [86, 159]. Shelter responses should be planned as part of an integrated system, where location, service access, and social conditions are prioritised alongside quality of physical structures, with coordination across shelter, health, and social sectors.

### Disaster preparedness role in mitigating poor health outcomes

A number of papers reviewed looked at the integration of emergency shelter management by public health bodies, finding that public health engagement before, during and after emergencies was beneficial[103, 115, 160]. In higher-resource settings such as China, this included the pre-allocation of public buildings as shelters and the co-location of basic medical services within these spaces, which improved access for vulnerable populations [103]. While such infrastructure may not be directly replicable in lower-resource or humanitarian contexts, the underlying principles which include; advance identification of safe shelter sites, integration of health services within shelter settings, and prioritisation of vulnerable groups are transferable. These approaches could be adapted using locally available structures and mobile or community-based health services to strengthen coordinated emergency responses in resource-constrained settings. Further, these findings support the inclusion and importance of public health bodies into disaster planning and preparation.

Public housing (state-owned) for disaster survivors in Japan were found to have higher rates of psychological distress, insomnia and PTSD [20, 119]. Contrary to this, disaster survivors living in rural locations or parks showed either no worsening of mental health scores or a lower prevalence of PTSD [57, 69]. While these findings are context-specific, they point to transferable considerations around the design of post-disaster shelter and relocation strategies. In particular, they suggest that factors such as social cohesion, familiarity of environment, overcrowding, and access to community support may play a critical role in mental health outcomes. For lower-resource and humanitarian settings, this highlights the importance of prioritising community-based or decentralised shelter approaches, preserving social networks where possible, and integrating mental health support into shelter planning, rather than focusing solely on the provision of formal housing structures.

Access to medicines and NCD care can be challenging following an emergency, when the majority of medical services focus more on communicable disease. Within the review, studies highlight how displacement to short-term and medium-term shelter impact on the management of chronic conditions [19, 46, 161]. Such findings underline the importance of maintaining continuity of NCD treatment during resettlement processes.

.

### Long term impact of shelter and settlement interventions on NCD

Results from this review indicate that post-disaster or displacement housing can have sustained effects on nutrition, diet, and physical activity, which in turn influence NCD risk. Short-term evidence from Türkiye shows that container housing can make it difficult to maintain NCD-specific nutrition [161], while longer-term studies from Japan demonstrate persistent unhealthy dietary habits, elevated cholesterol, and increased obesity among residents of temporary housing [116, 123, 162]. Similar patterns are seen for physical activity: in Algeria, 43% of people in a refugee camp reported low activity levels [66] and in Japan, obesity risk persisted for years after relocation to temporary housing [116, 117]. These results highlight how the design, location, and duration of temporary or emergency housing can directly influence long-term health behaviours. Context matters: factors such as space for cooking or exercise, access to healthy foods, social support, and proximity to services all shape outcomes. Even where the physical structure is adequate (e.g. in Japan), insufficient attention to these contextual elements may lead to long-term increases in NCD risk. For lower-resource or humanitarian settings, this suggests the need to integrate nutrition, physical activity, and health promotion considerations into shelter planning, alongside traditional protection and safety measures.

This review shows a critical gap in humanitarian programming where NCD care and risk prevention are seldom prioritised in shelter and settlement planning, yet housing type, location, and duration of displacement shape access to medicines, nutrition and lifestyle behaviour, which all impact NCD health outcomes. This suggests that humanitarian actors need to design shelter strategies that explicitly account for the continuity of NCD care, especially in view of the increasing burden in LMICs.

## Strengths and limitations

Our study has a number of limitations that should be considered. First, there is an absence of a clear or standardized framework for categorizing shelter interventions in humanitarian crises. This lack of conceptual clarity made it difficult to consistently classify interventions across studies and to compare their health impacts in a structured way. Shelter interventions were variably described in terms of accommodation type, quality of accommodation, duration of displacement, or service package, which limited the ability to draw meaningful comparisons or identify patterns in the data, and which lead to the development of the five themes used to present findings in this paper. This inconsistency also highlights the need for agreed-upon typologies to guide future research and program evaluation in this area.

Secondly, adopting a global lens in this review introduces important challenges. The evidence base spans highly heterogeneous contexts, ranging from sudden-onset natural disasters in high-income countries to protracted conflicts in settings such as Gaza Strip. These contexts differ significantly in terms of baseline infrastructure, governance, security, and population vulnerability. As a result, the effectiveness and feasibility of shelter interventions are deeply context-dependent. Also, the heterogeneity in study design, population groups, and health outcomes which range from communicable and NCDs to mental health and injuries limited the comparability of findings and precluded meta-analysis. This heterogeneity limits the direct comparability of findings and underscores the need for caution in generalizing results. It also highlights a critical gap in context-specific evidence from low-income and conflict-affected settings, where shelter needs are often greatest. Future research should prioritize these settings and explicitly examine how interventions can be adapted to account for resource constraints, insecurity, and prolonged displacement.

.

Third, only three electronic databases were searched, which raises the possibility that relevant studies indexed in other databases or grey literature sources were missed. A high proportion of papers included were cross sectional studies, which cannot establish causality and the potential for selection bias. The lack of shelter or settlement terms, equivalent to MeSH terms, limited the precision of database searches.

Lastly, only articles published in English were included, which may have excluded valuable insights from non-English publications, particularly those originating from regions frequently affected by humanitarian crises.

Despite these limitations, the wide variety of contexts considered and sectors of health analysed offers a unique view on the interaction between shelter and settlement interventions on health outcomes.

## Conclusions

This systematic review highlights that shelter and settlement interventions in humanitarian crises significantly influence a wide range of health outcomes including communicable and non-communicable diseases, mental health, injury risk, nutrition and overall well-being. The exact impact of shelter and settlement conditions on health outcomes varies by context, emphasising the need for context specific assessments to better understand the underlying drivers of health to better inform locally appropriate shelter solutions. This evidence synthesis emphasises the importance of considering shelter not just as a physical necessity but as a critical determinant of health shaped by housing condition, quality of shelter, social cohesion, proximity to services, familiarity of environment, and context-adapted shelter strategies. It emphasises the need for further research in this area, to help elucidate specific patterns across different types of hazard and populations.

A key contribution of this review is the identification of shelter as a core entry point for public health action, highlighting the need for clearer operational guidance on shelter typologies, minimum standards, and their integration with health, protection and social services. The findings also point to the potential for relatively low-cost shelter improvements to generate meaningful health gains, while underscoring the importance of considering cost-effectiveness, climate resilience, and long-term impacts in shelter design and planning.

Importantly, the review reveals critical gaps in current humanitarian programming, particularly the limited integration of mental health and NCD prevention and care into shelter responses, despite clear links between living conditions and long-term health risks. It also highlights the challenges of applying a global evidence base across diverse contexts, reinforcing the need for more research in low-income and conflict-affected settings where needs are greatest.

Furthermore, the evidence in this review points to the important role for public health leadership in disaster preparedness and shelter planning, alongside the need for clearer guidance on shelter typologies that promote mental well-being and ensure continuity of care. Shelter responses should be designed in close coordination with health systems to ensure uninterrupted access to essential services, particularly for people with chronic conditions. There is need for planning beyond immediate response, with investments in adaptable and durable shelter solutions that can better support long-term health needs in both urban and non-urban contexts.

## Supplementary Information

Supplementary Material 1

Supplementary Material 2

Supplementary Material 3

## Data Availability

All data generated or analysed during this study are included in this published article and its supplementary information files.

## Abbreviations

AXIS: Appraisal tool for cross -sectional studies
CASP: Critical appraisal skills program (CASP)
EHI: Essential household items
GBV: Gender-based violence
GEJE: Great East Japanese Earthquake
HH: Household
HIC: High income country
ITN: Insecticide-treated nets
LIC: Low income country
LMIC: Lower middle income country
LPG: Liquified petroleum gas
MAM: Moderate acute malnutrition
MCS: Mental component summary
MDE: Major depressive episode
MeSH: Medical subject headings
MetS: Metabolic syndrome
MUPS: Medically unexplained physical symptoms
NCDs: Noncommunicable diseases
NOS: Newcastle-ottowa scale
NRC: Norwegian refugee council
oPt: Occupied palestinian territories
PRISMA: Preferred reporting items for systematic reviews and meta-analyses
RCT: Randomised control trial
SAM: Severe acute malnutrition
SRH: Sexual and reproductive health
UMIC: Upper middle-income country

## Footnotes

1 Includes Hurricane, Tsunami, Typhoon, Cyclone,

2 self-reported health reports, included chronic illness (including hypertension, diabetes, back pain, asthma and heart problems, acute illness (including influenza, diarrheal diseases, food poisoning, infections, tonsillitis), mental health and disability (including blindness, deafness, paralysis, amputations and inability to speak.) and self reported physical, mental or psychological health outcomes.

3 Including mass location to higher ground, reconstruction on the same site or privately moving to a remote place.
