# Supplementary material for: The health outcomes of shelter and settlements interventions in humanitarian crises: a systematic review
Source: Confl Health. 2026 Jun 20;20:83. doi: 10.1186/s13031-026-00811-3 (PMC13625370; doi:10.1186/s13031-026-00811-3)
Supplement: Supplementary file 1 [file 13031_2026_811_MOESM1_ESM.docx]

**PROTOCOL**

**Evidence Synthesis: The health outcomes of shelter and settlements interventions in humanitarian crises - A systematic review protocol**

April 2025

# Table of Contents

**Table of Contents	2**

**Abbreviations	3**

1. Background and Rationale	4

2. Objectives	4

3. Research Question	5

Sub-questions	5

Definitions	5

4. Inclusion and Exclusion Criteria	6

5. Population	6

6. Interventions	7

7. Search strategy	7

Information Sources	7

Search Strategy	8

8. Study Selection	9

9. Data Extraction	9

10. Data Synthesis	10

Subgroup and Sensitivity Analyses	10

11. Discussion	10

12. Conclusion	10

13. Dissemination	11

14. Funding and Conflicts of Interest	11

# Abbreviations

| GSC | Global Shelter Cluster |
| --- | --- |
| NCDs | Noncommunicable diseases |
| GBV | Gender based violence |
| LIC | Low-income country |
| LMIC | Low-and-middle income country |

# 1.  Background and Rationale

Humanitarian crises, whether caused by conflict or disasters triggered by natural hazards, profoundly disrupt the lives of affected populations.^[1]^ Provision of shelter and formation of temporary settlements are one of the pillars of an emergency response, providing not just physical protection but also being an important contributor to health, well-being, and dignity.^[2]^ However, the evidence base on the complex and multifaceted links between shelter and health outcomes remain relatively underexplored in humanitarian contexts, limiting the ability of policymakers and practitioners to consider evidence based solutions.

We aim to consolidate existing research, identify knowledge gaps, and highlight key findings to better inform policy, advocacy, and humanitarian programming. This systematic review specifically focuses on examining the relationship between shelter, settlements, and health outcomes in humanitarian crises.

A key objective of the Global Shelter Cluster (GSC) Strategy is to integrate shelter and settlements interventions with other technical sectors. GSC Strategic Priority 1.1 aims to “Promote integrated approaches to preparedness and response,” with GSC strategic priority 2.3 aiming to increase coverage of shelter and settlement responses, which includes:

- Emphasizing that shelter is foundational to achieving broader humanitarian outcomes.
- Demonstrating the consequences of inaction in shelter provision.
- Promoting a multi-sectoral approach through linked or joint programming.^[3]^

# 2. Objectives

1. Mapping and analyzing existing evidence on the connections between shelter, settlements, and health in humanitarian crises.
2. Identifying gaps in this evidence base to inform future research priorities.
3. Presenting findings in actionable formats to support policy, advocacy, and practice.
4. Providing clear recommendations for further studies to strengthen understanding and inform interventions.

# 3. Research Question

How does shelter and settlement assistance influence health outcomes in humanitarian crises, and under what conditions do these impacts occur?

## Sub-questions

1. What are the specific health outcomes (e.g. communicable diseases, non-communicable diseases (NCDs), maternal health, mental health, gender-based violence (GBV), injuries, malnutrition) influenced by shelter and settlement assistance in humanitarian crises?
2. How do different types of physical structures and household items^[4]^ (e.g. blankets, stoves, tents, temporary shelters, prefabricated homes) affect physical and mental health outcomes in humanitarian crises?
3. What evidence is there of how shelter and settlement processes (e.g. co-creation, community participation) impact the health of individuals and communities in humanitarian crises?
4. How do shelter and settlement interventions such as site and settlement planning, the repair of physical structures including through retrofitting and upgrading etc. security of tenure and transfers including in-kind, vouchers, multi-purpose cash or “cash for shelter” assistance impact the health of affected populations?

## Definitions

**Humanitarian crises:** Humanitarian crises encompass man-made, non-man-made disasters triggered by natural hazards and pandemics specific to humanitarian settings.

**Shelter and settlement assistance:** A process of sheltering and associated neighbourhood and community interventions, which typically includes non-food items, plastic sheeting, tents, shelter kits, temporary or transitional shelters, technical support, rental and hosting support, collective centres, technical support including information and guidance and construction training. Shelter and settlement assistance extends to include repair of structures, retrofitting, upgrading, transfers, through in-kind, vouchers, multi-purpose cash or “cash for shelter”assistance and site and settlement planning^[5]^.

**Health outcomes:** Health outcomes refer to the impacts of shelter and settlement interventions on both physical and mental health, including communicable diseases, NCDs, GBV, maternal health, mental health disorders, malnutrition, injuries and conditions related to environmental conditions (e.g., hypo- and hyperthermia).

# 5. Inclusion and Exclusion Criteria

**Inclusion Criteria:**

- Primary research studies: Including observational studies, case reports, case series, case studies, retrospective studies and cohort studies with primary data that detail the health outcomes of shelter and settlement interventions in humanitarian settings.
- Literature Types: Peer-reviewed articles, grey literature (such as government and health organization reports, guidelines on construction/organization of shelter and settlements, reports of interventions, official documents from national health authorities, WHO, CDC, or similar organizations that provide data or evaluation)
- Population: Studies from humanitarian settings, with preference from low- and middle-income countries (LMICs).
- Time Frame: Publications from 2005.^[6]^

**Exclusion Criteria:**

- Language: Non-English articles unless a reliable translation is available.
- Data Quality: Studies with insufficient data or poor methodological quality that cannot provide actionable insights.
- Study type: conference abstracts, commentaries, letters to editors, posters, systematic reviews
- Population: Studies from high-income countries where there is no humanitarian crisis.
- Time frame: Publications before 2005

# 5. Population

The review will focus on shelter and settlement interventions for populations affected by humanitarian crises, prioritizing LMICs. It will also consider upper-middle-income countries where appropriate.

Key health outcomes will include:

- Communicable diseases, NCDs, GBV, mental health conditions, maternal care, injuries and environmentally induced conditions, such as hypo- and hyperthermia, malnutrition.
- The focus will be on the following types of humanitarian crises: man-made conflict; disasters triggered by natural hazards and climate change impact: e.g. extreme temperatures, fires, floods, dust storms etc.
- Displacement crises including refugee and internally displaced persons camps, host communities and residual population.

Key health outcomes will not include:

- The impact of healthcare facilities or water and sanitation infrastructure which does not form part of a habitable living space.
- Injuries sustained in workplaces.

# 6. Interventions

Shelter interventions in humanitarian settings. These could include but are not limited to non-food items, plastic sheeting, tents, shelter kits, temporary or transitional shelters, technical support, rental and hosting support, collection centres, technical support including information and guidance and construction training.

**Outcomes:**

- Effectiveness of Strategies: Mapping and analyzing existing evidence on the connections between shelter and settlement interventions and health in humanitarian crises.
- Challenges and Barriers: Identification of challenges to the successful implementation of shelter and settlement interventions in improving health outcomes in humanitarian settings.
- Gaps in Literature: Studies that highlight missing information, areas of uncertainty, or inconsistencies in the current research landscape.
- Policy and Future Recommendations: Research that offers recommendations for targeted shelter and settlement interventions which lead to evidence based improvement in health outcomes.

# 7. Search strategy

## Information Sources

- Electronic Databases: PubMed, Embase, Medline
- Grey Literature: Reports and publications from WHO, CDC, OCHA, Shelter Cluster, CCCM Cluster, Habitat for Humanity, IOM, ICRC, IFRC, UNHCR, Health Cluster, NRC, DRC.
- Year: from 2005

## Search Strategy

The choice of an evidence synthesis is to examine the scope and range of available literature on health outcomes related to shelter and settlement interventions in humanitarian crises.

The search strategy will include the following terms “noncommunicable disease” OR “communicable disease” OR “gender-based violence” OR “maternal health” OR “mental health” OR malnutrition OR hyperthermia OR hypothermia OR “parasitic disease*” OR “growth disorder*” OR "Pregnancy Complications" OR "Pregnancy"OR "Pregnant Women" OR “burns”OR “wounds and injuries” **AND** “natural disease*”, disaster, refugee, “refugee camp”, “internally displaced person”, “disaster victim*”, “disaster medicine”, “relief work”, war, “armed conflict*”, “humanitarian cris*”, “humanitarian emergenc*”, “forced migration”, Conflict-affected, War-affected, “Emergency Setting*", "Crisis Setting*", OR Catastroph* "large-scale emergency*", "mass emergenc*", Crisis, Conflict*, Hurricane,Tornado*, Flood*, “Storm Damage” OR earthquakes OR “volcanic activity” **AND** "Equipment and Supplies" OR "Disaster Planning" OR "Sheltered Housing" OR Shelter* OR House OR Housing OR Home* OR Dwelling* OR Settlement* OR Neighbourhood* OR Neighborhood* OR tent* OR “Local evacuation center” OR “Respite center” OR “Hurricane evacuation center” OR “Wildfire Evacuation center” OR “Family assistance center” OR “short term shelter” OR “Mega Shelter” OR “staff shelter” OR “overcrowding” OR “collective centre*” OR “collective center*” OR “transit camp*” OR “transit site*” OR “prefab*” OR “winterization” OR “winterisation” OR “communal shelter” OR “host family” OR “Cash and voucher assistance” OR “cash transfer programme” OR “CTP” OR “cash based interventions” OR “CBA” OR “cash based assistance” OR “CVA” OR “cash transfers” OR “cash-for-shelter” OR “cash for shelter” OR “in-kind” OR “cash and voucher delivery mechanisms” OR “cash and voucher” OR “rental payments” OR “rental support” OR “rental assistance” OR “minimum expenditure basket” OR “multi-purpose cash” OR “ host assistance” OR “ security of tenure”.

**The following key words will only be searched for in the title or abstract:** Conflict-affected, War-affected, “Emergency Setting*", "Crisis Setting*", OR Catastroph* "large-scale emergency*", "mass emergenc*", Crisis, Conflict*, Hurricane, Tornado*, Flood*, “Storm Damage”, earthquakes, “volcanic activity” Shelter*, House, Housing, Home*, Dwelling*, Settlement*, Neighbourhood*, Neighborhood*, tent*, “Local evacuation center”, “Respite center, “Hurricane evacuation center”, “Wildfire Evacuation center”, “Family assistance center”, “short term shelter”, “Mega Shelter”, “staff shelter” “overcrowding”, “collective centre*”, “collective center*”, “transit camp*”, “transit site*”, “prefab*”, “winterization”, “winterisation”, “communal shelter”, “host family”

**The following key words will be searched with medical subject headings (MeSH) terms in Pubmed:** “noncommunicable disease”, “communicable disease”, “gender-based violence”, “maternal health”, “mental health”, malnutrition, hyperthermia, hypothermia, “parasitic disease*”, “growth disorder*”, "Pregnancy Complications", "Pregnancy”, "Pregnant Women" “burns”, “wounds and injuries” “natural disease*”, disaster, refugee, “refugee camp”, “internally displaced person”, “disaster victim*”, “disaster medicine”, “relief work”, war, “armed conflict*”, "Equipment and Supplies", "Disaster Planning","Sheltered Housing".

**The following key words will be excluded:** "care home*", "medical home*", "nursing home*", "home care", "medical house", "home-grown", "home treatment", "home-based", "home-delivered", "home-produced" , "take-home", "at-home"

To capture different terms used for health outcomes of shelter and settlements in humanitarian crises, different search terms will be used. Boolean logic “OR” and “AND” and “NOT” will be applied. Adaptations to the above key terms will be made for Embase and Medline. A full search strategy can be found in appendix A.

# 8. Study Selection

The consultants (LAO and EJ) will conduct study selection based on predefined inclusion and exclusion criteria. Data will be extracted from the search results, and duplicates arising from searches across multiple databases will be removed using EndNote reference management software.

A two-phase screening process will be followed to determine which studies will be included in the final review. The first phase involves an initial screening of titles and abstracts to assess alignment with the inclusion criteria. Only studies meeting these criteria will be retained for the next phase. The second phase will involve a full-text review of the selected articles to confirm their eligibility for inclusion in the review.

Screening will be conducted using the Rayyan systematic review platform. References from EndNote will be imported into Rayyan to facilitate the screening process. Both reviewers (LAO and EJ) will independently assess the studies, and their final selections will be compared. In cases where discrepancies arise and cannot be resolved through discussion, a third reviewer (JB) will be consulted to reach a consensus.

During the first phase, an inclusion/exclusion form will be used to document decisions based on title and abstract review. If eligibility remains unclear at this stage, the full text of the publication will be retrieved for further assessment.

# 9. Data Extraction

Once the authors have reached consensus on the studies to be included in the review, a tailored data extraction form will be used to collect key information such as the first author, year of publication, title, study design, study population, and study setting. Data extraction will be carried out by the investigators LAO and EJ, with JB verifying the entries for accuracy. The extracted data will align with the review’s objective of examining the relationship between shelter and settlement interventions and health outcomes in humanitarian settings. Specific data points will include: type of shelter or settlement intervention implemented; characteristics of the population targeted; health outcomes reported (e.g., communicable diseases, NCDs, mental health, GBV, maternal health, malnutrition); contextual factors such as displacement type, crisis type, and setting; and implementation features including design, materials, duration, safety, ventilation, privacy, and accessibility.

**Quality Assessment**

The quality of all included studies will be assessed using tools appropriate to each study design.

- Observational studies will be evaluated using the Newcastle-Ottawa Scale, which assesses aspects such as selection, comparability, and outcome reporting.
- For randomized controlled trials, the Cochrane Risk of Bias Tool will be used to examine potential sources of bias across key domains.
- Qualitative studies will be appraised using the Critical Appraisal Skills Programme (CASP) checklist, focusing on clarity of aims, methodological appropriateness, and the rigor of data collection and analysis.
- Grey literature, including reports and guidelines, will be assessed using the Flinders University AACODS checklist, which evaluates Authority, Accuracy, Coverage, Objectivity, Date, and Significance.

# 10. Data Synthesis

After consensus of inclusion has been completed, a tailored data extraction form will be used to collect key information such as the first author, year of publication, title, study design, study population, and study setting. Data extraction will be carried out by the investigator LAO, with EJ verifying the entries for accuracy. The extracted data will align with the review’s objective of examining the relationship between shelter and settlement interventions and health outcomes in humanitarian settings. Specific data points will include: type of shelter or settlement intervention implemented; characteristics of the population targeted; health outcomes reported (e.g., communicable diseases, NCDs, mental health, GBV, maternal health, malnutrition); contextual factors such as displacement type, crisis type, and geographic setting; and implementation features including shelter design, materials used, duration of stay, safety, ventilation, privacy, accessibility, and service delivery modality (e.g., cash, in-kind, voucher).

Additionally, data will be extracted on reported challenges or barriers to implementation, enabling conditions for success, and any recommendations or lessons learned related to the intervention. These variables will support the analysis of intervention effectiveness, implementation challenges, gaps in the literature, and policy-relevant findings.

## **Subgroup and Sensitivity Analyses**

A sub-group analysis will be performed on the type of shelter or settlement intervention implemented during different phases of humanitarian response (emergency, recovery, and reconstruction). This is because shelter interventions vary depending on the response phase, and these differences may influence health outcomes. For example, emergency shelters such as tents and plastic sheeting may address immediate protection needs but differ in impact from transitional shelters or permanent housing used during recovery and reconstruction. Additional sub-group analyses will be conducted based on the category of health outcome (e.g., communicable diseases, NCDs, mental health, GBV), the geographic setting (e.g., country or continent), the type of humanitarian crisis (e.g., conflict, disasters triggered by natural hazards, climate-related), and the population served (e.g., refugees, IDPs, host communities). These sub-group analyses will help identify variations in findings across contexts and inform more nuanced recommendations for policy and practice.

# 11. Discussion

The findings of this review will be interpreted in relation to existing literature on shelter and health outcomes in humanitarian settings, highlighting how the evidence contributes to current understanding and identifying its implications for public health policy, clinical practice, and humanitarian programming. Particular attention will be given to how the results support or challenge prevailing assumptions and practices. The discussion will also address the limitations of the review, including potential gaps in the evidence base, methodological constraints, and publication bias. Areas where further research is needed to strengthen the evidence base and inform more effective shelter and settlement interventions will be clearly outlined.

# 12. Conclusion

The final review will present a concise summary of the key findings, highlighting the connections between shelter and settlement interventions and health outcomes in humanitarian settings, and emphasizing their relevance for future program design. Based on the evidence gathered, the review will offer actionable recommendations to inform policy development, strategic planning, and implementation for the GSC and its partners. These recommendations will address both operational and research priorities, supporting more integrated, multi-sectoral evidence-based shelter responses that contribute meaningfully to improved health outcomes in crisis-affected populations.

# 13. Dissemination

To ensure wide dissemination and impact, the review will be submitted for publication in a peer-reviewed journal. In addition to journal publication, the final review report will be shared on the GSC webpage to reach operational and policy audiences. Opportunities should also be explored to present key insights at relevant forums such as the Global Health Cluster and GSC meetings, to inform practice and encourage uptake of the findings across humanitarian sectors

# 14. Funding and Conflicts of Interest

- Disclose any funding sources or potential conflicts of interest.

1. Townes D, ed. In: Health in Humanitarian Emergencies: Principles and Practice for Public Health and Healthcare Practitioners. Cambridge University Press; 2018:iii-iii.
2. <https://emergency.unhcr.org/emergency-assistance/shelter-camp-and-settlement/shelter-and-housing/emergency-shelter-solutions-and-standards>
3. GSC Strategy 2024-2028, Shelter. Life saving, Life enabling, Shelter Cluster, 2024.
4. Including mattresses, blankets, clothes, wind-proofing, mosquito nets and excluding WASH and food items.
5. Description of humanitarian shelter and settlements sector activities, Bureau for democracy, conflict, and humanitarian assistance and the office of U.S foreign disaster assistance, USAID, 2019
6. 96% of humanitarian papers identified in previous studies were found to be from after 2005. The wider impacts of humanitarian shelter and settlement interventions. Annex B Methodology. Interaction USAID, 2020.
