# Supplementary material for: The health outcomes of shelter and settlements interventions in humanitarian crises: a systematic review
Source: Confl Health. 2026 Jun 20;20:83. doi: 10.1186/s13031-026-00811-3 (PMC13625370; doi:10.1186/s13031-026-00811-3)
Supplement: Supplementary file 2 [file 13031_2026_811_MOESM2_ESM.docx]

Appendix 2

# 2.1 Methodology

**Protocol Development**

Following the Preferred Reporting Items for Systematic Reviews and Meta-Analyses (PRISMA) guidelines, a protocol was formulated for this review. Its objective was to examine the relationship between shelter, settlements, and health outcomes in humanitarian crises. The protocol was developed between the end of March to 17 May 2025 by a team of three consultants (EJ, LA, JB) and was reviewed by the steering committee for the Global Shelter Cluster (Appendix 1.1). Following final discussion the systematic review aimed to address the following question and subquestions:

**Question:**

How does shelter and settlement assistance influence health outcomes in humanitarian crises, and under what conditions do these impacts occur?

**Sub-questions:**

1. What are the specific health outcomes (e.g. communicable diseases, non-communicable diseases (NCDs), maternal health, mental health, gender-based violence (GBV), injuries, malnutrition) influenced by shelter and settlement assistance in humanitarian crises?
2. How do different types of physical structures and household items^[1]^ (e.g. blankets, stoves, tents, temporary shelters, prefabricated homes) affect physical and mental health outcomes in humanitarian crises?
3. What evidence is there of how shelter and settlement processes (e.g. co-creation, community participation) impact the health of individuals and communities in humanitarian crises?
4. How do shelter and settlement interventions such as site and settlement planning, the repair of physical structures including through retrofitting and upgrading etc. security of tenure and transfers including in-kind, vouchers, multi-purpose cash or “cash for shelter” assistance impact the health of affected populations?

**Literature search**

An extensive literature search was carried out across three databases—Embase (via Ovid), PubMed, Medline. Three persons conducted the search between 16 - 21 April 2025 to identify relevant studies related to the review topic. The search strategy was developed using a combination of keywords and Medical Subject Headings (MeSH) terms related to shelter, settlement, and health. Boolean logic operators “OR” and “AND” were employed to refine the search, and truncations were used for terms like “humanitarian cris*”, “humanitarian emergenc*”, Catastroph* (See Appendix 1.1 for full search strategy).

For this systematic review, any peer- reviewed study with primary data collection that reported on shelter or settlement interventions and their impact on health outcomes in humanitarian settings, published between January 2005 to April 2025 and published in English was included. 2005 was chosen as the start of the search because 96% of humanitarian papers identified in previous studies on shelter and health outcomes were found to be from after 2005 [(1)](https://web.endnote.com/citations/eyJkaXNwbGF5VGV4dCI6IigxKSIsImNpdGF0aW9ucyI6W3siZ3VpZCI6IjNkYWJmMzExLWM2OWItNDZmZS1hMWZmLTRlMjhiOWI3N2E2YSIsInJlY29yZCI6eyJkYXRlcyI6eyJ5ZWFyIjoiMjAyMCJ9LCJ0aXRsZXMiOnsidGl0bGUiOiJUaGUgd2lkZXIgaW1wYWN0cyBvZiBodW1hbml0YXJpYW4gc2hlbHRlciBhbmQgc2V0dGxlbWVudCBpbnRlcnZlbnRpb25zLiIsInNlY29uZGFyeS10aXRsZSI6IkludGVyYWN0aW9uIn0sImNvbnRyaWJ1dG9ycyI6eyJhdXRob3JzIjp7ImF1dGhvciI6IkludGVyYWN0aW9uIn19LCJyZWMtZ3VpZCI6IjNkYWJmMzExLWM2OWItNDZmZS1hMWZmLTRlMjhiOWI3N2E2YSIsInJlZi10eXBlIjoiMTciLCJyZWMtdXNuIjoiNDE3In0sImJpYmxpb0NvbnRlbnQiOlt7InNlY29uZGFyeVRpdGxlIjoiSW50ZXJhY3Rpb24iLCJ5ZWFyIjoiMjAyMCIsInRpdGxlIjoiVGhlIHdpZGVyIGltcGFjdHMgb2YgaHVtYW5pdGFyaWFuIHNoZWx0ZXIgYW5kIHNldHRsZW1lbnQgaW50ZXJ2ZW50aW9ucy4iLCJncm91cEd1aWRzIjpbXSwicmVjb3JkU3RhdHVzIjoiYWN0aXZlIiwiZ3VpZCI6IjNkYWJmMzExLWM2OWItNDZmZS1hMWZmLTRlMjhiOWI3N2E2YSIsInJlZmVyZW5jZVR5cGUiOiIxNyIsInJzeG1sIjoiPHJlY29yZD48cmVmLXR5cGU%2BMTc8L3JlZi10eXBlPjxjb250cmlidXRvcnM%2BPGF1dGhvcnM%2BPGF1dGhvcj5JbnRlcmFjdGlvbjwvYXV0aG9yPjwvYXV0aG9ycz48L2NvbnRyaWJ1dG9ycz48dGl0bGVzPjx0aXRsZT5UaGUgd2lkZXIgaW1wYWN0cyBvZiBodW1hbml0YXJpYW4gc2hlbHRlciBhbmQgc2V0dGxlbWVudCBpbnRlcnZlbnRpb25zLjwvdGl0bGU%2BPHNlY29uZGFyeS10aXRsZT5JbnRlcmFjdGlvbjwvc2Vjb25kYXJ5LXRpdGxlPjwvdGl0bGVzPjxkYXRlcz48eWVhcj4yMDIwPC95ZWFyPjwvZGF0ZXM%2BPHJlYy1ndWlkPjNkYWJmMzExLWM2OWItNDZmZS1hMWZmLTRlMjhiOWI3N2E2YTwvcmVjLWd1aWQ%2BPHJlYy11c24%2BNDE3PC9yZWMtdXNuPjwvcmVjb3JkPiIsImF1dGhvcnMiOlsiSW50ZXJhY3Rpb24iXX1dfV19). Additionally, grey literature detailing the impact of shelter or settlement interventions on health outcomes in humanitarian settings were included. Exclusion criteria included systematic reviews, commentaries, editorials, opinion pieces, general narrative reviews, conference abstracts and research protocols. Studies from high-income countries (HIC) where there is no humanitarian crisis were also excluded. Inclusion and exclusions can be seen in table 1.

**Table 1: Study inclusions and exclusions**

| Inclusions for the systematic review | |
| --- | --- |
| Variables | Humanitarian setting  Shelter or settlement intervention  Health outcomes |
| Language | English |
| Date range | January 2005 to April 2025 |
| Paper type | Peer review publications |
| Exclusions for the systematic review | |
| Paper types | systematic reviews, commentaries, editorials, opinion pieces, general narrative reviews, conference abstracts and research protocols |

**Data Extraction and Synthesis**

Retrieved articles were imported into Endnote for initial duplicate removal. Remaining records were then uploaded into Rayyan, where additional duplicates were eliminated. Two reviewers independently screened the titles and abstracts, followed by full-text screening. Any disagreements during the screening process were resolved through consultation by the two reviewers. A standardized data extraction form was used to collect key information from the included studies, such as study characteristics (author, publication year, and study design), type of crises, population, country, displacement type, shelter intervention, settlement interventions, duration of displacement, description of intervention, health outcome. Some papers reported more than one crises and more than one health outcome.

Additionally, data was extracted on reported challenges or barriers to implementation, enabling conditions for success, and any recommendations or lessons learned related to the intervention.

**Analysis**

Data was examined to identify key themes. These were documented using excel and summarised on powerpoint for discussion with the steering group.

First, the extracted data was reviewed and categorised by key study characteristics (Appendix 2).

Data collected on shelter and settlement intervention and health outcome variables were reviewed and categorised into 5 emerging themes (living condition, type of accommodation, camp and site planning, shelter and settlement status, shelter assistance).

Within each theme, the data was further grouped into sub-groups and data of the subgroups was synthesised into tables.

**Report**

All shelter and settlement interventions are reported using the same terminology as used in the journal articles. All geographical locations are referred to using the same terminology of the journal article.

1. Including mattresses, blankets, clothes, wind-proofing, mosquito nets and excluding WASH and food items.
