# Supplementary material for: The health outcomes of shelter and settlements interventions in humanitarian crises: a systematic review
Source: Confl Health. 2026 Jun 20;20:83. doi: 10.1186/s13031-026-00811-3 (PMC13625370; doi:10.1186/s13031-026-00811-3)
Supplement: Supplementary file 3 [file 13031_2026_811_MOESM3_ESM.docx]

**Appendix 3**

Contents

[Theme 1: Living conditions	1](#_Toc226371700)

[1.1	Housing Conditions	1](#_Toc226371701)

[1.2	Overcrowding	10](#_Toc226371702)

[1.3	Energy	15](#_Toc226371703)

[Theme 2: Accommodation type	18](#_Toc226371704)

[2.1	Short term shelter	18](#_Toc226371705)

[2.2	Medium term shelter	23](#_Toc226371706)

[2.3	Long term shelter	28](#_Toc226371707)

# Theme 1: Living conditions

## 1.1 Housing Conditions

| **Author** | **Country** | **Humanitarian Event** | **Population** | **Shelter variable** | **Health variable** | **Results** |
| --- | --- | --- | --- | --- | --- | --- |
| Haq, 2023 | Pakistan | Flood affected | Affected populations, < 5 years | Mud, stone, concrete and brick houses | Diarrhoea | Housing children in mud houses were found to be 2.5 times more likely to have diarrhea than those in stone, concrete, or brick houses (OR = 2.54, P < 0.05). |
| Deka, 2021 | India | Flood | Affected population, < 12 years | Flood affected housing with at least 1 flood per year | Soil transmitted helminth (STH) infection | -Building material: Concrete vs earthen floor showed no statistically significant difference in STH  Flood prone houses: increased odds of all STH infections (OR 2.2; CI 1.3–2.6, p = 0.004), particularly hookworm (OR; 2.8, CI 1.4–5.9, p = 0.005) |
| Takarinda, 2022 | Zimbabwe | Displacement, armed conflict | Refugees | Damage to housing | Malaria | Holes in housing/open eaves: odds of contracting malaria were 3.37 times higher for those residing inside. |
| Semakula, 2023 | Uganda, refugee settlement | Displacement | Refugee, < 5 years | Building material | Malaria | Building material: HH whose walls were constructed using thatch and cardboard were 4.50 (aOR; 2.50, CI: 0.11–56.0) and 2.30 (aOR; 2.30, CI: 1.39–13.2) times more likely to have their children contracting malaria compared to households whose walls were built with brick and cement or mud (p < 0.05).  -Households with thatch roofs were 2.12 (aOR; 2.12, CI: 0.94–4.79) times more likely to have children contracting malaria than those with roofs constructed of tarpaulins and iron sheets. |
| Searle, 2023 | Madagascar | Cyclone | Affected population, < 18 years | Damaged housing | Malaria | Malaria prevalence: higher in HH with significant damage (35.8%; 24.9–49.1) or complete destruction (44.2%; 30.5–58.7) compared to households with no damage (22.1;13.9–30.0) |
| Habib, 2014 | Lebanon, camps or ‘gatherings’ | Displacement | Refugees |  | Multi-morbidity | Water leakage: Nearly twice as likely to have three or more health reports (OR: 1.88, CI: 1.45–2.44) than the baseline.  -More pronounced among respondents living in households with severe food insecurity. |
| Cardoso, 2016 | Cote d'Ivoire | Armed conflict, displacement | IDPs, Host communities | House, quality of housing | GBV | -“We live in a poor neighborhood and the way the houses are built…everyone is aware of everything that happens to you.” (Female, IDP)  -“We are in urban areas here. We are living in common courtyard houses. In these kinds of houses, you can’t really have a private life. The houses are so close together and the courtyard is common, so everybody will hear what’s happened in your household. If you husband batters you all the time, everybody will know and you’ll no longer be respected by the neighbors. Even their children will disrespect you. That’s why we are so concerned with “other’s eyes.” (Female, IDP) |
| Balakrishnan, 2024 | Kenya, informal settlements | EWE | Affected populations | Housing structures; galvanized iron sheets, mud, wood, concrete, narrow, unpaved paths, minimal vegetation. | Physical health  Mental health | Sleep disturbances and fatigue: Reported by participants and fatigue across multiple EWEs, including flooding, heatwaves, downpours, and increased mosquitos.  Stomach issues: Reported during bad air events  Respiratory: Reported during heat waves, colder-than-usual temperatures, extreme wind, and bad air quality events.  Other: Chest pain and tightness, high blood pressure, pain, skin issues, such as rashes and bites, general discomfort and restlessness. They described mental health impacts, such as worry, stress and anxiety. |
| Hamamra, 2025 | Gaza, oPt | Armed conflict | IDPs | Community shelters | Mental health  Sleep disturbances | -“We sleep in shifts now,” explained one mother, “because we can’t all close our eyes at the same time. Who will watch over us?” In these overcrowded spaces, sleep is rationed out like food, taken sparingly and guarded. Another resident described their experience as “sleeping with one eye open,” alert to every sound, ready to flee at a moment’s notice. The crowded shelters have robbed Gaza’s residents of the safety and peace that sleep should bring, leaving them in a state of constant wakefulness and heightened anxiety.  -One resident shared, “My body is giving up. I feel tired all the time, but I can’t rest; sleep has become a foreign thing.” Sleep deprivation in Gaza leads to chronic exhaustion, with individuals reporting constant fatigue, impaired concentration, and heightened irritability. “I feel my body weakening,” one woman states, “and my mind is foggy; it’s like I’m slowly fading.” For these individuals, the lack of sleep is not merely a nightly inconvenience but a constant, cumulative threat to their well-being. |
| Andrade, 2021 | Puerto Rico | Hurricane | IDPs | Private shelters | Unmet health needs | unsatisfactory living conditions (refuges) were described as contributors to declining older adult physical and emotional health, especially for those with complex medical needs |
| Smeeth, 2022 | Lebanon | Displacement, armed conflict | Refugees, 8-16 years | Tent | PTSD; CPSS^[1]^, Hair cortisol concentration | C[urrent living conditions were not associated with hair cortisol concentration](https://pmc.ncbi.nlm.nih.gov/articles/PMC9908541/#Tab3) |
| Pérez-Sales, 2022 | Greece | Displacement | Refugees | Reception centre | Suicidal ideation    Psychological suffering | -Bathroom and personal hygiene: difficulties of access, lack of hygiene, and lack of privacy.  -> 90% of the camp was an unhealthy space full of garbage, producing or aggravating diseases derived from them. To this, they added the impact of the psychological suffering of having to live in this environment.  -94% of those interviewed have had suicidal thoughts.  -99% of respondents reported difficulties in carrying out activities of daily living |
| Tay, 2015 | Papua New Guinea | Armed conflict, displacement | Refugees | Non permanent shelters | Mental disorder | -37% of participants were forced live in poor conditions due to ongoing violence  -Housing condition was classed as one traumatic event.  -Traumatic event count showed a dose-response pattern in its relationship with functional impairment, the greatest risk (AOR=11.47; 95% CI=2.11-62.37) being for those experiencing the highest level of traumatic event exposure (16-23 events). |
| Akinyemi, 2012 | Nigeria, refugee camp | Displacement, armed conflict | Refugees, host communities, migrants | Size of house | Mental health | -Poor mental health: associated with living in a one bedroom accommodation (OR = 0.82, CI = 0.35 - 1.38, p=0.295) |
| Sitwat, 2015 | Pakistan | Flood | Affected population, 13-19 years | Tent, house | PTSD  GAD  MDD; DSM-IV-TR c^[2]^ | -Classified diagnosis: Highest in subgroup of most damage to house.  -High levels of classified diagnoses in each subgroup make interpretation difficult. |
| Thapa, 2011 | Nepal | Armed conflict | IDPs, adults |  | Depression; HSCL-25  Anxiety; HSCL-25 ^[3]^  PTSD; PCL-C^[4]^  Disability; WHO-DAS II^[5]^ | -Disability: Lack of housing was positively associated with disability. (p=0.04)  -Mental health: No statistical significant results for anxiety, depression or PTSD |
| Karaki, 2021 | Lebanon | Displacement | Refugees | Not mentioned | NCDs | -“You'd have to walk on a mountain of garbage to get [to the house].” (36 F, Syrian Refugee (SR) with six children)  -Most SRs raised concerns about living conditions in the camp, including a lack of clean water and garbage collection, high unemployment, and high rent.  -80-83% of respondents reported rats / mice / mosquitos / cockroaches  -SRs reported NCDs such as hypertension, diabetes, asthma, and back pain; mental health issues (e.g., stress, anxiety, and depression); pediatric illnesses; injuries; and pregnancy-related concerns. Some cited their environment and life in the camp as the main factors contributing to their illness.  -SRs described surrounding areas as dirty, with piles of uncollected garbage, the smell of sewage, and rodent and cockroach infestations. |
| Brandenberg, 2006 | USA | Hurricane | Affected population | Evacuation shelter | Injuries | -Identification of dangers such as chemicals, choking hazards in emergency shelter  -Reduction in number of injuries to 1 from 3 |
| Sabah, 2025 | Gaza, oPt | Armed conflict | IDPs, 25-65 years | Damaged buildings | Respiratory disease  General health | -"Our house was damaged during the last war, and since then, we have no proper windows or doors. The wind and dust come in all the time, and my children are constantly sick with respiratory problems"  -Key informants confirmed that damaged housing infrastructure, coupled with limited access to repair services, was a primary factor contributing to poor health outcomes in Gaza. |
| Al-Khatib, 2005 | West Bank, oPt | Armed conflict, Displacement | Refugees | Damaged buildings | General health  Mental health | 97% of women agreed that there is a great effect of housing conditions on health and well-being.  -A third of those interviewed could see that the old building with cracked walls and roofs was an unhealthy house, and this affected their health indirectly they suffered from fear of collapse of their old house, which caused stress, anxiety and loss of sleep |
| van Zandvoort, 2022 | Somalia | Displacement, Food insecurity | IDPs | Shelters made of corrugated sheets | Respiratory diseases; direct and indirect contacts | Respiratory disease: No evidence of any increase in the odds of cumulative incidence of self-reported pneumonia diagnosis for household quality.^[6]^ |

## 1.2 Overcrowding

| **Author** | **Location** | **Type of crisis** | **Population** | **Shelter variable** | **Health variable** | **Result** |
| --- | --- | --- | --- | --- | --- | --- |
| Ul Huq, 2023 | Pakistan | Flood affected | Affected populations, < 5 years | Mud, stone, concrete and brick houses | Diarrhoea | HH size: significant association, with children in HHs with a large number of people (above 8) being about 7 times more likely to have diarrhea than those in HHs with 1–4 people (OR = 7.46, p < 0.01). |
| van Zandvoort, 2022 | Somalia, IDP camp | Displacement, food insecurity | IDPs | Shelters; corrugated sheets | Respiratory diseases; direct and indirect contacts | Respiratory disease: No evidence of any increase in the odds of cumulative incidence of self-reported pneumonia diagnosis for HH size, members nor the total number of contacts.^[7]^ |
| Logie, 2021 | Uganda, informal settlements | Displacement, armed conflict | Refugees |  | Barriers to HIV testing | ‘There are other factors that should also be looked at like privacy and confidentiality. When you look at refugees, you will find that 90-95% of refugee families live in slums. You might find a family of eight or nine people living in one room, a husband, wife and seven children, because of the poor living standards, they can't afford like a house whereby the children will be living in their separate rooms, so privacy and confidentiality at home will hinder that. In organisations like (blinded), we can provide a room and shade for self‐testing’. |
| Habib, 2014 | Lebanon, camps and ‘gatherings’ | Displacement | Refugees | Concrete buildings | Multi-morbidity | -Crowded HHs: 21% (OR: 1.21, CI: 1.04–1.41) more likely to report two poor health outcomes and 27% (OR: 1.27, CI: 1.02–1.57) more likely to report three health problems than homes without crowding. |
| Mazhak, 2024 | Czechia | Displacement | Refugees | Room in a shared house | Perceived stress, PSS-10 | -Lack of living space: Associated with increasing level of perceived stress (β=0.11 for PSS-10), living with strangers : Associated with increase levels of stress (β=1.42) |
| Ugbe, 2023 | Nigeria, host communities | Displacement | IDPs in host communities | Overcrowded shelters | Somatoform disorder | -Living in overcrowded shelters: significant relationship between overcrowded shelters and developing the somatoform disorder in the study population, b = 0.84, p = 0.008, aOR = 2.30 (CI: 1.25, 5.26), |
| Jabali, 2025 | West Bank, oPt, camps and non-camps | Armed conflict | Affected population | Houses, camps | Physical, mental and psychological health; ^[8]^ | -Number of people living in a HH: 4 -6 individuals in a HH reported the highest mean physical health score (M: 3.85, SD: 0.96), closely followed by those who lived in smaller homes with one to three members (M: 3.79, SD: 1.05).  -Number of people living in a HH: 4-6 Camp Individuals in HH had the greatest mean score for mental health (M: 3.79, SD: 1.02); those in HHs with 1–3 members also reported better mental health outcomes (M: 3.8, SD: 1.1).  -Number of people living in a HH: 4-6 individuals in HH mean score for psychological health was greatest (M: 3.55, SD: 0.91) for families with 4–6 persons, and it was closely followed by those in smaller homes (M: 3.53, SD: 1.14). |
| Ismail, 2020 | Lebanon, camps | Displacement | Refugees | Tent, temporary shelters | Food security | -Registration and assistance: Syrian refugees with assistance were found to have a significantly higher crowding and total number of children compared to other groups (p = 0.002 and p = 0.001).  -Food security: majority of unregistered Syrian refugees and those registered without assistance were found to be severely food insecure compared to refugees receiving assistance (83.3 vs 78.6 vs 43.1%, respectively), p<0.001. |
| Abi Chahine, 2022 | Lebanon, refugee camps, host communities | Displacement, armed conflict | Refugees, Host communities |  | Communicable diseases | “Many of the older refugees’ physical health problems are caused by lack of hygiene, access to water, and malnutrition (…). Sometimes, 30 people live in the same tent, children and young adults go to school or to work and rarely bathe. This causes skin diseases like scabies. As for malnutrition, often a family would only have bread and tomatoes as a meal. Older adults are more sensitive to malnutrition which diminishes their immune system making them less able to fight even a simple flu or diarrhoea.”  “…older refugees suffer more frequently than younger refugees from pulmonary diseases in winter due to lack of heating and to overcrowding whereby viruses spread rapidly among the residents of the same tent or house.” |
| Doocy, 2013 | Haiti, Camp and non-camps | Earthquake | Affected populations, Camp and no camp populations |  | Injury rates | -Crowding: significant impact on rate of injury (p = 0.014), with HHs with between 2.0 and 2.9 individuals per room experienced significantly higher injury rates than those with fewer than 2.0 people per room and more than 3.0 people per room.  Camp living: significantly higher injury rate than those in neighbourhoods at 46.7/1000 as compared to 33.7/1000 (p = 0.018).  Individuals from HHs with 2.0–2.9 people per room were 1.44 (CI: 1.03–2.03, p = 0.03) more likely to experience injury than those with < 2.0 people per room, and residents of camps were 1.44 (CI: 1.06–1.95, p = 0.02) times more likely to experience injury than neighbourhood residents. |
| Lett, 2006 | Uganda | Displacement, Armed conflict | Refugees, IDPs | Temporary or semi- permanent accommodation | Injuries | Risk of injury: fewer rooms in the house were related to injury  -Number of rooms: showed an OR of 0.968, and therefore a small decrease risk in injury for each additional room, of 3.25% |
| Karaki, 2021 | Lebanon, | Displacement | Refugees | Not mentioned | NCDs | -Some HCWs attributed NCD burden to SR's low socioeconomic status and overcrowded living conditions.  -Most SRs described crowded living conditions with families of five or more living in one to two rooms. SRs described surrounding areas as dirty, with piles of uncollected garbage, the smell of sewage, and rodent and cockroach infestations. |

## 1.3 Energy

| Author | Location | Humanitarian event | Population | Shelter variable | Health variable | Outcome |
| --- | --- | --- | --- | --- | --- | --- |
| Abu Ibaid, 2021 | Gaza & West Bank, oPt | Displacement | IDPs, Affected population | Gas heater  Fires | Carbon monoxide poisoning | “Winter is the time when families sit crammed in one room around the gas heater or the burned wood; it is a huge risk especially when you have children and elderly” (CHW, Khan Younes).  “People die from inhaling fumes resulting from closing all the doors and windows, while keeping the heater on” (CHW, Hebron).“During the winter, children tend to ignite more fires in the backyard of the house; they like to think that is for heating, but it is more for plying” (Mother, Jordan Valley). |
| Pérez-Sales2022 | Greece | Displacement | Refugees | Shelter quality | Thermal comfort | All of the interviewees expressed having suffered extreme temperature conditions, both cold and heat and humidity conditions, without the possibility of protecting themselves against them. Temperatures in summer reach 40 ◦C and in winter can reach 5 ◦C at night, with moderate rainfall. The building structures were minimal and generally dedicated to the sanitary or service area. Only the area for unaccompanied children had a concrete structure |
| Mori, 2007 | Japan | Earthquake | Affected population | Shelter quality | MSK pain | Patients with rheumatism were aware that cold temperatures could have a deleterious effect on their physical condition. Some patients experienced knee pain during times of low ambient temperature; in some instances, their knees became so swollen that they could not wear trousers or they had difficulty walking down stairs |
| Yaksi, 2024 | Türkiye | Earthquake | Host communities, IDPs | Access to heating | PTSD; CDI, PTSD-RI, SCARED, CYRM^[9]^ | Access time to heating: not being met with 72 hours p=0.01, significantly associated with probable severe PTSD  -Inadequate meeting of heating needs in the 5th month. p value = 0.152. significantly associated with probable severe PTSD |
| Giusti, 2022 | Italy | Earthquake | Affected populations | Heating | Viral illness | -“I always had a sore throat, in short, always a little cold ... because it was cold, the tent was damp, the heating was not enough.” |
| Presser, 2024 | Lebanon | Displacement | Refugees | Heating | Injuries | -Using electricity for heating was found to be the safest method, with closed and open flames being associated with more injury (p<0.01). -Having a child lock on the heating was associated with a reduction in the risk of burns (p=0.000). |
| Matsubara, 2014 | Japan | Earthquake, Tsunami | Affected population | Gas supply | Depression | Depressive reaction associated with the unavailability of gas supply (p = 0.002) |
| Matsubara, 2016 | Japan | Earthquake, Tsunami | Affected population | Electricity and gas supply | PTSD | Access to water, electricity and gas showed no statistical significance |
| Ruggiero, 2012 | USA | Hurricane | Affected population | Access to electricity | PTSD; ^[10]^  MDE,  Anxiety; ^[11]^ | Participants reported being without electricity for an average of 13.0 days (SD = 22.9)  Lack of electricity for > 1 week: Association with PTSD, and MDE (p=0.00). No statistical significant association with GAD |
| Andrade, 2021 | Puerto RIco | Hurricane | Affected population | Electricity cuts | NCDs  Mental health | Electricity cuts lasting weeks to months: Lengthy power outages were also reported by participants as severely affecting mental health and presenting significant long-term challenges for the refrigeration of medicines like insulin. |
| Nöthling, 2023 | South Africa | Flood | Affected population | Loss of infrastructure | Anxiety; GAD7  Emotional Distress; K6  Depression; CES-D^[12]^  PTSD, HTQ^[13]^ | -75.4% lost electricity for at least 1 to 7 days  -Loss of infrastructure, including electricity: predictor of post-flood emotional distress and anxiety |
| Simeone, 2023 | Puerto Rico | Hurricane | Affected population | Electricity cuts | Postpartum depression (PPD) | Nearly all women cited at least 1 adverse hurricane experience (99.3%); 97.1% indicated losing electricity for at least 1 week; insufficient data to run against PPD |
| Richardson2023 | Greece, Serbia, Macedonia, Türkiye, France, the Netherlands, Belgium | Displacement | Refugees, migrants | Access to heating and shade | Thermal comfort | “I was there in the summer so it was hot, there’s not enough shading and people couldn’t find a place outside the sun. It’s more horrible now, as its winter, so it’s freezing, and there’s no heating. To be honest it’s worse than the refugee camps I’ve seen in Africa”  “They are a human rights violation. I don’t think people should be living in them, especially in a first world European country this should not be happening. People are living in cold tents without heating and electricity, there’s rats, there’s scabies, there’s a lack of toileting facilities, a lack of hygiene, lack of food.” |

# Theme 2: Accommodation type

## 2.1 Short term shelter

| **Author** | **Location** | **Humanitarian event** | **Population** | **Shelter intervention / variable** | **Health variable** | **Outcomes / finding** |
| --- | --- | --- | --- | --- | --- | --- |
| Lin, 2015 | Taiwan | Typhoon | Affected population | Community shelters compared to displaced within the community | Cases of infectious diseases | Seroconversion to Entomoeba histolytica: significantly higher in community shelter group compared to displaced within community (56.1% vs 36.8%; p=0.001)  Seroconversion to Rubella: significantly higher in community shelter group compared to displaced within community (OR=2.055, 95% CI 1.251 to 3.374; p=0.004).  Cases of mumps: shelters had a significantly higher rate (81.6% vs 69.5%, p=0.022)  Cases of HSV-2: shelters had a significantly lower rate of HSV-2: 27.2% vs 40.2%, p=0.023) |
| Yee, 2007 | USA | Hurricane | Affected population | Superdrome | Outbreaks | Norovirus outbreak |
| Balasuriya, 2024 | USA | Floods | Affected populations | Living in travel trailers, campers, cottages, campgrounds | NCDs | Overall health: Interviewees stated worsening of allergies (41%), high blood pressure (36%), overall health (32%), asthma (31%), COPD (22%).  Medication access: 18% reported difficulty getting prescriptions |
| Bilgehan, 2025 | Türkiye | Earthquake | Affected populations | Tent cities | Access to medications and care | Medication need: those in tent cities had significant decrease in proportion of participants able to meet their medication needs compared to pre-earthquake, 50% decrease, (p<0.05)  Access to hospitals: those living in tent cities 60% decrease compared to pre-earthquake  NCD-specific nutrition: 62% decrease, compared to pre-earthquake  -Receive adequate social support: 19 % decrease compared to pre-earthquake |
| Nakano, 2016 | Japan | Earthquake | Affected populations | Evacuation centre | MSK pain | -Musculoskeletal pain: significantly greater proportions of shelter use. |
| Jinnouchi, 2020 | Japan | Earthquake | Affected population | Community shelters | MSK pain | -Compared to absent pain, both participants with prevalent and exacerbated musculoskeletal pain showed significantly greater proportions of shelter use |
| Wang, 2010 | China | Earthquake | Affected population | Community shelters, house | Physical health; PCS^[14]^ | -Living in a temporary shelter: negatively associated with PCS (OR -5.04, 95% CI -7.68 to 2.39), |
| Lett, 2006 | Uganda | Displacement, Armed conflict | Refugees | Temporary accommodation / tents | Injuries | -Rate of injuries: Temporary housing linked to increased risk of injury. -Proportion of injury victims: Half the injury victims were IDPs 75% of injury victims were crowded in temporary shelter |
| Basaran, 2024 | Türkiye | Earthquake | IDPs | Community shelters, tents | Injuries | -Day 0-7 after earthquake: 84% of injuries occurred at the residential home  -Day 8-45 after earthquake: tents were the most common location for injuries (47.8%). Many outdoors in open areas.  -Day > 45 after earthquake:tents were the most common location for injuries (38.1%). Many outdoors in open areas.  -Nearly half of all burn injuries were scalds, with a third being flame burns, particularly in temporary shelters such as community shelters, tents, and prefabricated structures.  -More unstable shelters had the highest rates of electrical burns. |
| Chowdhury, 2022 | Bangladesh | Cyclone | Affected populations, women | Cyclone shelters | GBV | -Women living in community shelters often face unique and compounded challenges, including physical and mental abuse, as well as GBV |
| Albelbeisi, 2025a | Gaza, oPt | Armed conflict | IDPs, adults | Tents, community shelters (schools) | Depression  Anxiety  Stress; DASS21^[15]^ | -Mental health outcomes: Improved in those sheltering in community shelters (schools), compared to tents, mean stress scores of 32.21 vs. 30.11 (P = 0.016), anxiety scores of 31.80 vs. 29.29 (P = 0.014), and depression scores of 31.87 vs. 29.27 (P = 0.004). |
| Borah, 2025 | India | Flood | Affected population, adults | Relief camps, embankments or roads, homes of relatives | PTSD; PCL-5^[16]^  Anxiety; PHQ-4  Depression; PHQ-4^[17]^ | -Prevalence of mental health: no significant difference in prevalence of mental health conditions between participants who were displaced and those who were not |
| Kun, 2009 | China | Earthquake | IDPs, > 12 years | Temporary shelters, portable homes compared to original homes | PTSD, HTQ and DSM-IV^[18]^ | -PTSD: higher in individuals living in temporary shelter or houses (OR=3.18; 95% CI=2.07, 4.89) |
| Erdoğan, 2024 | Türkiye | Earthquake | IDPs | Tents and containers | PTSD; PCL-C^[19]^ | -PTSD score/presence of PTSD: Significantly higher in individuals staying in tents than those staying in other places (p < 0.001) |
| Yaksi, 2024 | Türkiye | Earthquake | Host communities IDPs | Tent, Container, Permanent housing | PTSD;CDI, PTSD-RI, SCARED, CYRM^[20]^ | -Rates of probable severe PTSD: no significant association between tent, container or apartment |
| Albelbeisi, 2025b | Gaza, oPt | Armed conflict | IDPs | Tents, community shelter, house | Malnutrition; SAM, MAM, NNS | -Accomodation: Those living in a house had the highest rates of SAM and MAM ( 12.8% SAM, 15.4% MAM); living in a tent (5.5% SAM, 8.8% MAM) p value= 0.001. living in a school had the lowest (4.1% SAM, 7,3% MAM), living in a tent 5.5% SAM, 8.8% MAM p value= 0.001; children living in houses had an increased likelihood of MAM (OR = 1.44; 95% CI = 1.13–1.84; p = 0.003) and SAM (OR = 2.36; 95% CI = 1.39–3.99; p = 0.001)  -SAM: 31.5% lived in houses, 49.7% in schools, and 18.8% in tents.  -NNS: highest among school residents (65.0%), followed by those in houses (23.0%) and tents (12.0%). |
| Mir Mohamadalile2019 | Iran | Earthquake | Affected population, breastfeeding women | Tents | Breastfeeding | -Barriers: lack of a safe and private environment for infants’ feeding |
| Brandenberg, 2006 | USA | Hurricane | Affected population, 5-18 years | Evacuation shelter management | Injuries and burns | -Identification of dangers such as chemicals, choking hazards in emergency shelter  -Reduction in number of injuries to 1 from 3 |
| Ishii, 2011 | Japan | Earthquake | Affected population | Emergency shelter management | Communicable diseases | -Two contamination-control certified nurses at the hospital visited each emergency shelter  -Contamination-control certified nurses were sent to the shelters where there was an increase in the number of patients with symptoms |
| Wu, 2019 | China | Flood | Affected population | Emergency shelters (schools), management | Communicable diseases  Chronic diseases | -Prebuilt shelters were found to allow the provision of water, food, gas and electricity and were felt to be a more stable accommodation than tents.  -Mass sheltering also made it easier to conduct concentrated health management  -Half of shelter points had temporary medical points; pregnant women and newborns were priority transferred.  -Township governments made final decisions on sheltering sites but consulted with public health personnel regarding public health. |

## 2.2 Medium term shelter

| **Author** | **Location** | **Humanitarian event** | **Population** | **Shelter intervention / variable** | **Health variable** | **Results / Outcome** |
| --- | --- | --- | --- | --- | --- | --- |
| Takahashi, 2020 | Japan | Earthquake | Affected population, adults | Home compared to prefabricated houses (similar to FEMA trailer homes) | Obesity  MetS | MetS: Highest in temporary housing (coeff.: 0.20; CI 0.01 to 0.43]; p = 0.045), with differences in cholesterol^[21]^, and obesity, but not HbA1C or blood pressure. The increase in MetS was especially true for women.  Obesity and BMI: No significant difference  Cardiovascular risk factors: Higher prevalence of current smokers in temporary housing, low physical activity and poor dietary intake. No significant difference in < 65 years |
| Yazawa, 2023 | Japan | Earthquake, Tsunami | Affected population, 65-89 years | Temporary trailer homes built by the city (kasetsu jutaku), subsidized apartments in the private rental market (minashi kasetsu), or new houses, prefabricated housing, rented accommodation | Dietary patterns; DHQ ^[22]^ | Dietary patterns: Those living in trailer-style temporary housing had lower consumption of high intake of a diet consisting of alcoholic beverages, meat, and seafood, compared to those who did not move, 9 years after the earthquake |
| [Kuniyoshi](https://pubmed.ncbi.nlm.nih.gov/?term=Kuniyoshi+Y&cauthor_id=31156072),2019 | Japan | Earthquake | Affected population, < 5 years | Prefabricated housing units, public-funded rental housing and public housing | Eczema  Respiratory disease | Shelter type: Prevalence of eczema in prefabricated temporary housing higher than for children living in other types of housing. (OR, 1.46; 95% CI,1.06–2.02; p = 0.022)  The same was not seen with respiratory diseases (OR, 0.97; CI 0.61–1.54; p = 0.88). |
| Nagata, 2015 | Japan | Earthquake, Tsunami | Affected population, > 20 years | Temporary housing complexes | General health; self-report of general health status  Psychological distress; K6^[23]^ | General health: “Fair” or “poor” in 31% at 21-23 months; higher nationwide sample in Japan of 10–30%. No significant difference between 10-12 months and 21-23 months.  Psychological distress: Prevalence of 43.5% with K6 scores above the cut-off point, higher than general Japanese population of 27.5% |
| Nakano, 2018 | Japan | Earthquake, Tsunami | Affected populations, > 20 years | Temporary housing, rental housing | Smoking status | Starting smoking after the disaster: Increased individuals who lived in temporary housing (PR1.46; CI: 1.10 to 1.93), and those in rental housing had a PR of 1.38 ( CI: 1.16 to 1.64)  -Changes in smoking statuses were associated with disaster-associated psychosocial factor |
| Akaishi, 2021 | Japan | Tsunami | Affected population, adults | Prefabricated houses | Psychological stress, K6,  Depression; CES-D^[24]^  PTSD; IES-R^[25]^  Insomnia; AIS ^[26]^ | Mental health outcomes: History of living in temporary prefabricated housing did not worsen scores in the 5 psychosocial or psychiatric measures 5 years after the disaster. Improvement in AIS, but not statistically significant. |
| Morishima, 2020 | Japan | Earthquake | Affected population, adults | Prefabricated houses, privately-rented temporary housing | Psychological distress, K6  Sleep disturbances | Psychological distress: Living in prefabricated (β = 0.046, p = 0.031) and privately-rented temporary housing (β = 0.043, p = 0.042) predicted later psychological distress; Residents in prefabricated temporary housing scored significantly higher on psychological distress than those in their own home (p < 0.001); living in temporary housing at was not related with psychological distress at year 4, but predicted psychological distress at year 5.  Sleep disturbances: no mediation effect by sleep disturbances (prefabricated temporary housing: β = 0.001, p = 0.620; privately-rented temporary housing: β = −0.001, p = 0.467) |
| Kaplan, 2024 | Türkiye | Earthquake | Affected population, adults | Tent, container | Somatic conditions  Paranoia  Psychosis  Anxiety | Somatization: Living in a container predicted lower somatization (β = −0.757), paranoid ideation (β = −0.609), and psychosis (β = −0.610), but higher anxiety (β = 1.161) and phobic anxiety (β = 0.475) |
| Tanji, 2018 | Japan | Earthquake | Affected populations, adults | Prefabricated houses | Psychological distress, K6 | -Psychological distress was significantly higher in those who among participants who had lived in prefabricated temporary housing for >4 years, compared to than those who had lived there for <3 years.  - Among participants who had shown lower psychological distress at the baseline, the proportion of those who showed deterioration of psychological distress was significantly higher when they had lived in prefabricated temporary housing for >4 years than when they had lived there for <3 years. |
| Kawakami, 2020 | Japan | Earthquake, tsunami | Affected population, adults | Temporary housing shelters | Seeking healthcare  MDE; case numbers  PTSD; case numbers | Mental health: proportions of individuals who visited psychiatrists or other physicians among people with post-disaster were greater in the shelter group (42.6%, 26 of 61 cases) than in the general population (17.4%, 4 of 23 cases)( p = 0.011).  MDE: proportions seeking treatment for MDE were 39.5% (17 of 43 cases) for the shelter group and 30.8% (4 of 13 cases) for the general population ( p = 0.339).  PTSD: proportion of treatment for PTSD (9.1%) was lower than for the other disorders in the shelter group |
| Lu, 2020 | China | Earthquake | Affected populations | CRS | PTSD; PCL-C^[27]^ | Moving to CRS: Lower prevalence for symptoms of PTSD. (OR, 0.58; 95% CI, 0.34–0.97) |
| Suzuki, 2014 | Japan | Earthquake | Affected population | Reconstructed on the same site, disaster recovery public condominium, mass-translocation to higher ground, privately moving to remote areas | Psychological distress; K6  Depression; CES-D^[28]^  Insomnia; AIS^[29]^  PTSD; IES-R^[30]^ | Depression: Scores of the CES-D were significantly higher, indicating higher distress among those who moved into disaster-recovery public condominiums. (p < 0.0001)  Insomnia: not different among the four residential types  Psychological distress/PTSD: The scores in the public condominium group were slightly higher, but not statistically significant (p = 0.0345, p = 0.0281) |
| Sasaki, 2017 | Japan | Earthquake | Affected population, > 65 years | Prefabricated houses, existing private accommodation, newly established housing, house | Depression; GDS^[31]^ | -Participants who moved into new accommodations were 1.5 times more likely to report depressive symptoms than those who did not move (adjusted rate ratio = 1.51, 95% CI: 1.14–2.00).  -Moving into prefabricated housing had a 30% stronger association with depressive symptoms than other types of relocation (aRR = 2.07, 95% CI: 1.45–2.94). |
| Ide-Okochi, 2022 | Japan | Earthquake | Affected population, adults | Prefabricated temporary housing, temporary housing in the private or public rented sectors, rented housing, public housing, post-disaster public housing, hospitals, retirement homes, and other types of accommodation. | Depression, Psychological distress; K6  Insomnia, AIS^[32]^  PTSD; PDS^[33]^ | Psychological distress: Participants with rented housing (OR = 1.50, CI = 1.21–1.87), public housing (OR = 2.14, CI = 1.63–2.83), public housing for disaster survivors (OR = 2.16, CI = 1.08–4.32), were more likely to experience psychological distress.  Insomnia: more likely to be associated living alone (OR = 1.20, CI = 1.04–1.38), public housing (OR = 1.54, CI = 1.26–1.88), loneliness (OR = 5.55, CI = 4.90–6.30),  Probable PTSD :more likely to be associated with rented housing (OR = 1.95, CI = 1.41–2.69), public housing (OR = 2.41, CI = 1.62–3.58), post-disaster public housing (OR = 2.75, CI = 1.23–6.16), no change in school district (OR = 2.07, CI = 1.01–4.28) |

## 2.3 Long term shelter

| **Author** | **Location** | **Humanitarian event** | **Population** | **Shelter intervention /variable** | **Health variable** | **Results / Outcomes** |
| --- | --- | --- | --- | --- | --- | --- |
| Albelbeisi, 2025b | oPt, Gaza^[34]^ | Armed conflict | IDPs, 24–59  months | House rental vs house ownership  City/ camp/ village | Anaemia | -Renting: Higher proportion of children with anemia l (24.7%) compared to children without anemia (14.3%).  -Owning a home: more common among families whose children did not have anemia (85.7%) versus those who did (75.3%). (p < 0.05). |
| Ide-Okochi, 2022 | Japan | Earthquake | Affected population | Rental housing, public housing, RPH | Hypertension compliance | Hypertension non-compliance: Rental housing (aOR = 1.92, 95% CI 1.20–3.07), public housing (aOR = 2.47, 95% CI 1.38–4.42) were associated |
| Jabali, 2025 | West Bank, oPt | Displacement, Armed conflict | Affected population | Private housing, Private flats | Physical health score;  Mental health score  Psychological health score | Housing type: Those living in private houses had greatest mean PHS (M: 4.24, SD: 0.56), followed by those living in private apartments (M: 4.16, SD: 0.61).  -Resided in their own houses and private flats had the best mean MHS (M: 4.14, SD: 0.72)(M: 4.15, SD: 0.6).  -Lived in private houses had the highest mean score (M: 3.78, SD: 0.79) for psychological well-being, and private apartments showed similar psychological well-being (M: 3.84, SD: 0.7). |
| Kinoshita, 2022 | Japan | Earthquake | Affected population | Disaster housing complexes, non housing complex group | Wellbeing; PGCMS^[35]^ | **The factors significantly related with the PGCMS scores for both groups were “poor health condition” (housing complex group β = −0.222, non-housing complex group β = −0.263),** “difficulties resting while asleep” (housing complex group β = −0.185, non-housing complex group β = −0.229), |
| Ide-Okochi, 2023 | Japan | Earthquake | Affected population | Prefabricated temporary housing, temporary housing private/ public rented sectors, rented housing, public housing, post-disaster public housing, hospitals, retirement homes, other | Psychological distress; K6  PTSD ; PDS^[36]^  Insomnia; AIS^[37]^ | Psychological distress: female gender (OR 1.33; CI 1.13–1.57), rented housing (OR 1.50; CI 1.21–1.87), public housing (OR 2.14; CI 1.63–2.83), public housing for disaster survivors (OR 2.16; CI 1.08–4.32) were more likely to experience psychological distress. No association with changing school district.  PTSD: female gender (OR = 1.81, 95% CI = 1.41–2.32), age of 65 years or above (OR 1.49; CI 1.16–1.90), rented housing (OR 1.95; CI 1.41–2.69), public housing (OR 2.41; CI 1.62–3.58), post-disaster public housing (OR 2.75; CI1.23–6.16), no change in school district (OR = 2.07, 95% CI = 1.01–4.28). |
| Muir, 2019 | Indonesia | Volcanic eruption | Affected population |  | Mental health | -They found that those who were moved on (compared to moved home or in transition (temporary housing)) were more likely to report good mental health, compared to average or poor, (proportional OR for moved home: 2.02 CI 1.05-3.91; pOR in transition: 2.02 CI 1.00-4.14) when modeled using a variety of demographic and disaster related variable |

1. Child PTSD Symptom Scale
2. Diagnostic and Statistical Manual of Mental Disorders, Fourth Edition, Text Revision
3. Hopkins Symptom Checklist-25
4. PTSD Checklist-Civilian Version
5. WHO Disability Assessment Schedule 2.0
6. Insufficiently weighed for reliable result
7. Insufficiently weighed for reliable result
8. The questionnaire structure includes three domains: Physical Health Outcomes, Mental Health Outcomes, and Psychological Well-Being Outcomes. Ten items per domain are used to evaluate the impact of housing on health indicators. Respondents rate their agreement using a five-point Likert scale.
9. Children’s Depression Inventory, Children’s Posttraumatic Response Reaction Index, Screen for Child Anxiety Related Emotional Disorders, Child, Youth Resilience Measure and Social Support Appraisals Scale for Children
10. National Women’s Study (NWS) PTSD module
11. Structured Clinical Interview for DSM-IV (SCID-IV)
12. Center for Epidemiologic Studies Depression Scale
13. Harvard Trauma Questionnaire
14. Physical component score
15. Depression, Anxiety and Stress Scale - 21 Items
16. PTSD Checklist-Civilian Version
17. Patient Health Questionnaire for depression and anxiety and the Post-Traumatic Stress Disorder Checklist for PTSD.
18. Harvard Trauma Questionnaire and DSM-IV criteria were used to diagnose PTSD
19. PTSD Checklist-Civilian scale
20. Children’s Depression Inventory, Children’s Posttraumatic Response Reaction Index, Screen for Child Anxiety Related Emotional Disorders, Child, Youth Resilience Measure and Social Support Appraisals Scale for Children
21. Breakdown of HDL, LDL and triglycerides in the study.
22. Brief Dietary History Questionnaire,
23. In the K6, participants who scored 5 points or more were at risk for psychological distress;
24. Center for Epidemiologic Studies Depression Scale
25. Impact of Event Scale-Revised
26. Athens Insomnia Scale
27. PTSD Checklist-Civilian Version (PCL-C).21 This 17-item self-administered questionnaire is based on Diagnostic and Statistical Manual of Mental Disorders, Fourth Edition (DSM-IV) diagnostic criteria B, C, and D for PTSD
28. Center for Epidemiologic Studies Depression Scale
29. Athens Insomnia Scale
30. Impact of Event Scale-Revised
31. Geriatric Depression Scale
32. Athens Insomnia Scale
33. Posttraumatic Diagnostic Scale
34. Study conducted before 2023 war.
35. Lawton’s Philadelphia Geriatric Center Morale Scale
36. Posttraumatic Diagnostic Scale
37. Athens Insomnia Scale
